# Relationship between meteorological factors, air pollutants and hand, foot and mouth disease from 2014 to 2020

**DOI:** 10.1186/s12889-022-13365-9

**Published:** 2022-05-17

**Authors:** Hongxia Peng, Zhenhua Chen, Lin Cai, Juan Liao, Ke Zheng, Shuo Li, Xueling Ren, Xiaoxia Duan, Xueqin Tang, Xiao Wang, Lu Long, Chunxia Yang

**Affiliations:** 1 Department of Epidemiology and Health Statistics, West China School of Public Health and West China Fourth Hospital, Sichuan University, No.16, Section 3, Renmin south road, Wuhou District, Chengdu, Sichuan China; 2 Department of Microbiology Laboratory, Chengdu Municipal Center for Disease Control and Prevention, Chengdu, Sichuan China; 3 Department of Gastroenterology, West China School of Public Health and West China Forth Hospital, Sichuan University, Chengdu, Sichuan China; 4 Non-Communicable Diseases Research Center, West China-PUMC C.C. Chen Institute of Health, Sichuan University, Chengdu, Sichuan China; 5 Department of Immunization Planning, Chengdu Municipal Center for Disease Control and Prevention, Chengdu, Sichuan China

**Keywords:** Hand, foot, and mouth disease, Meteorological factors, Air pollutants, Vaccination, COVID-19

## Abstract

**Background:**

Meteorological factors and air pollutants have been reported to be associated with hand, foot, and mouth disease (HFMD) epidemics before the introduction of vaccine. However, there is limited evidence for studies with long-term dimensions.

**Methods:**

We collected the daily HFMD counts, weather and air pollution data from 2014 to 2020 in Chengdu. Distributed lag non-linear models (DLNM) were used to assess the associations of meteorological factors and air pollutants on HFMD cases.

**Results:**

From 2014–2020, high relative humidity and precipitation and extremely high and low levels of PM_10_, O_3_, SO_2_ and CO increased the risk of HFMD. In pre-vaccination period, extreme high and low temperatures, PM_10_ and NO_2_, low precipitation and high concentrations of PM_2.5_ and O_3_ significantly increase the risk of HFMD; In post-vaccination period, high relative humidity and low level of CO can significantly increase the incidence of HFMD; During the period of COVID-19, only low temperature will significantly increase the risk of HFMD; Low concentration of air pollutants has the greatest impact on the 6–14 age group, while the high concentration of air pollutants has the greatest impact on the 0–1 age group.

**Conclusions:**

Our study suggest that high relative humidity and precipitation and extremely high and low levels of PM_10_, O_3_, SO_2_ and CO increased the risk of HFMD from 2014 to 2020. The results of this study provide a reference for local authorities to formulate intervention measures and establish an environment-based disease early warning system.

**Supplementary Information:**

The online version contains supplementary material available at 10.1186/s12889-022-13365-9.

## Introduction

Hand food and mouth disease (HFMD) poses a substantial burden to health in mainland China and there is no specific treatment for it. During 2013 to 2016 prior to the introduction of Enterovirus 71 (EV71) vaccine, the incidence and mortality of HFMD have been leading the type C notifiable infectious diseases, affecting an average of more than 2.2 million children every year in China (http://www.nhc.gov.cn/). Three inactivated monovalent EV71 vaccines had been licensed in China in 2016, the efficacy against EV71-associated HFMD reached 98.8%. Nevertheless, there is no consensus on the cross-protection of vaccines against non-EV71-associated HFMD [1–4]. Although vaccination has been promoted on a large scale after 2016, the average number of reported cases of HFMD in China still remains high during the period from 2017 to 2019, about 2 million per year, dominated by coxsackievirus A16 (CVA16), coxsackievirus A6 (CVA6) and coxsackievirus A10 (CVA10) (http://www.nhc.gov.cn/). Whereas the pandemic of Coronavirus disease 2019 (COVID-19) further complicates the situation. During the pandemic, the incidence of most notifiable infectious diseases in China showed a downward trend, including HFMD [5–7].

Environmental factors, such as temperature, relative humidity, wind speed, precipitation, and air pollutants, have been reported to pay an important role in the transmission of HFMD before the introduction of vaccine [8–11]. Climatic factors have been recognized to have effects on the reproduction of the virus, while pollutants are thought to affect the susceptibility of individuals resulting in increased number of HFMD cases [11–13]. A role for vaccination in these associations was hypothesized in previous study, which speculate that the human defense mechanism may regard some particles as a virus, and virus vaccination may have a beneficial effect on these particles [14]. Several epidemiological studies have also demonstrated that vaccine might modify the adverse effects of pollutants on some disease [14, 15]. In addition, due to COVID-19, governments have imposed restrictions on the movement of people, vehicles, and suspended industrial activities, resulting in a significant reduction in pollution levels [16]. Accordingly, we postulated that the relationship between environment factors and HFMD may be different in pre-vaccination, post-vaccination or COVID-19 epidemic periods. The evidence of the positive association between HFMD and environment factors have been cumulated these years. However, the relationship between environmental factors and HFMD after the introduction of vaccine need to further prove. Therefore, we analyzed the incidence of HFMD and environmental factors from 2014–2020.

## Material and methods

### Study area

Chengdu is the capital city of Sichuan, located in the Sichuan Basin in Southwest China, has a subtropical monsoon humid climate. The geographical location is between 102°54 ′ – 104°53 ′ E and 30°05 ′ – 31°26 ′ N.

### Data sources

We retrieved the daily counts of HFMD cases from the infectious disease surveillance systems of Chengdu Center for Disease Control and Prevention (CDC) from January 1, 2014 to December 31, 2020. Considering that the vast majority of HFMD cases involve children, this study only includes patients below 15 years old, which account for 99.32% of the total number of cases. The number of people vaccinated for each dose of EV71 vaccine in Chengdu were extracted from the Immunization Planning Information Management System at the Chengdu CDC. And we collected daily data of meteorological data, including temperature, relative humidity, wind speed and precipitation and air pollution indicators, namely, PM_2.5_, PM_10_, O_3_, SO_2_, CO and NO_2_ from the publicly accessible China National Weather Data Sharing System and the Sichuan Environmental Monitoring Center, respectively.

### Statistical analyses

According to previous studies [13, 17, 18], we used a quasi-Poisson regression model combined with distributed lag nonlinear models (DLNM) to estimate the effects of meteorological factors and air pollutants on HFMD incidence accroding to the basis of data distribution and the relationship between variables.

Yt = α + NS( X; df; lag; df) + NS (Time; df) + ΣNS (Xi) + βDOW_t_ –––- [13]

where α is an intercept and Yt refers to the daily counts of HFMD cases onset on day t; NS reference a natural cubic spline modeling the nonlinear lagged relationship between meteorological factors or air pollutants and HFMD incidence; X is the examined meteorological or air pollution variables that we want to research; Xi represents the several other meteorological and air pollution variables that should be controlled due to their modifying effect on HFMD incidence [13].

In this model, degrees of freedom (df) was 4 for mean temperature, relative humidity, wind speed, precipitation, SO_2_, PM_2.5_, PM_10_, NO_2_, CO and O_3_, as well as lag spaces with 3 df based on the Akaike information criterion for quasi-Poisson (Q-AIC); Accordingly, the maximum lag days were set to 14 according to the incubation period and previous studies [13, 19]. Time is the indicator variable used to control long-term trends and seasonality; and DOW stands for day of week. We set df = 7 for time variable because 7 per year has been justified as a balance between providing adequate control for seasonality and other confounding by trends in time, while leaving sufficient information from which to estimate exposure effects [20]. In this study, the effects of extreme meteorological factors and air pollutants on HFMD were examined and presented as relative risk (RR) by comparing 5th and 95th percentiles with their median values.

Subgroup analysis was conducted by gender(male and female) and age group (≤ 1y, 2–3y, 4–5y, 6–14y). The criteria for age grouping are based on differences in the outdoor activities and environmental exposures of the children belonging to different age groups [13].

Sensitivity analyses were conducted to control the co-linearity of vaccination and environmental factors by adding vaccination population to the model.

## Result

### Summary statistics for HFMD cases, meteorological factors, and air pollutants

A total of 315,441 HFMD cases were included in our study, including 182,331 of males and 133,110 of females, and the male-to-female ratio is 1.37:1. Table 1 presents the daily average number of HFMD cases was 125 from January 2014 to December 2020. The peak age of HFMD is 2–3 years old, the daily average number of cases was 65 and the maximum number was 410. Of the three time periods (Table.S1, S2, S3), pre-vaccination period had the highest average incidence of 141 cases per day and the maximum number of HFMD cases is up to 784. After the introduction of the vaccine, an average of 475 people were given the first dose every day, and 401 people received the second dose.

**Table 1:** Description of HFMD cases and Vaccination number in Chengdu from 2014 to 2020

			Mean ± SD		Min	P25	Median	P75	Max
Cases		Total	125 ± 112	1		48	104	165	780
	Gender	female	53 ± 47	1		20	44	71	337
		male	73 ± 65	1		28	61	96	452
	Age	≤ 1	33 ± 37	1		13	24	38	291
		2–3	65 ± 56	1		26	55	65	410
		4–5	24 ± 20	1		8	19	35	145
		6–14	7 ± 7	1		2	5	10	49
		≥ 15	2 ± 2	1		1	1	2	14
	Year	2014–2016	124 ± 74	8		60	121	174	392
		2017–2020	126 ± 135	1		37	94	151	785
		2020	74 ± 96	1		3	23	104	359
vaccination	Dose	1	475 ± 427	1		70	415	856	1464
		2	401 ± 371	0		58	211	739	1291I

Note:* SD *represent standard deviation, *Px *represent percentile of the data

See as Table 2, from 2014 to 2020, the average values of temperature, relative humidity, wind speed, precipitation, PM_2.5_, PM_10_, O_3_, NO_2_, SO_2_ and CO were 18.14 °C, 3.74 m/s, 75.45%, 2.89 mm, 60.27ug/m^3^, 91.92ug/m^3^, 74.10ug/m^3^, 51.47ug/m^3^, 10.88ug/m^3^ and 0.89 mg/m^3^, respectively. Of the three time periods (Table.S1, S2, S3), the average relative humidity was the highest in the period after vaccination, in addition, the average value of others meteorological factors were the highest in COVID-19 period.

**Table 2:** Description of environmental factors in Chengdu from 2014 to 2020

Variables	Mean ± SD	Min	P25	Median	P75	Max
temperature (°C)	18.14 ± 7.28	1.39	11.39	18.89	24.44	32.33
Wind speed (m/s)	3.74 ± 1.58	0.98	3.00	3.00	4.16	15.02
Relative humidity (%)	75.45 ± 11.35	28.11	67.80	75.95	84.15	99.87
Precipitation (mm)	2.89 ± 10.59	0	0	0	1.02	188.47
PM_2.5_(ug/m^3^)	60.27 ± 40.52	3.60	35.50	48.09	72.93	371.21
PM_10_(ug/m^3^)	91.92 ± 63.08	8.64	47.52	75.20	117.63	555.45
O_3_(ug/m^3^)	74.10 ± 39.30	1.86	42.55	68.50	111.12	178.48
NO_2_(ug/m^3^)	51.47 ± 20.75	2.07	37.12	49.28	64.44	126.49
SO_2_(ug/m^3^)	10.88 ± 9.18	0	5.46	7.71	13.56	76.42
C0(mg/m^3^)	0.89 ± 0.42	0	0.61	0.81	1.07	2.90

Note:* SD* represent standard deviation, *Px *represent percentile of the data

### Time series analysis for HFMD cases, meteorological factors, and air pollutants

**Fig. 1:**
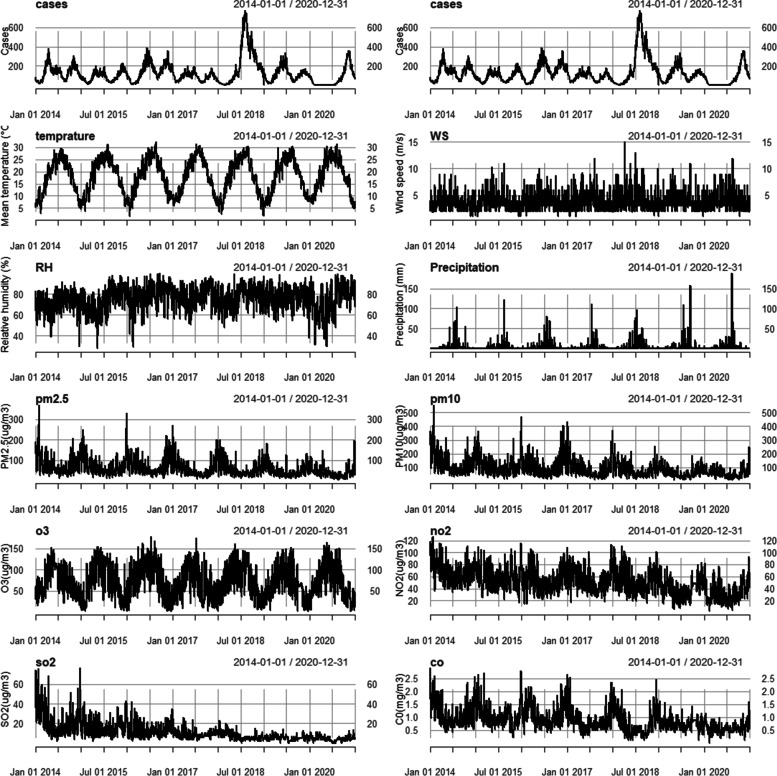
Time-series results of HFMD incidence and environmental factors in Chengdu from 2014 to 2020. WS: wind speed; RH:relative humidity

The time-series analysis illustrates the trends in daily HFMD cases and environmental factors from 2014 to 2020. We observed a significant seasonal variation for total cases, temperature, precipitation, PM_2.5_, PM_10_ and O_3_ from Fig. 1. Two significant peak was observed during late spring and early summer (from May to July) and late autumn and early winter (from October to December) except 2018 and 2020.

### Distributed lag non-linear models

We use the overall picture to visualize the effect for different meteorological and air pollutants variables at different lag days. All 3-D graph of the relative risk (RR) compared with their median value.

**Fig. 2:**
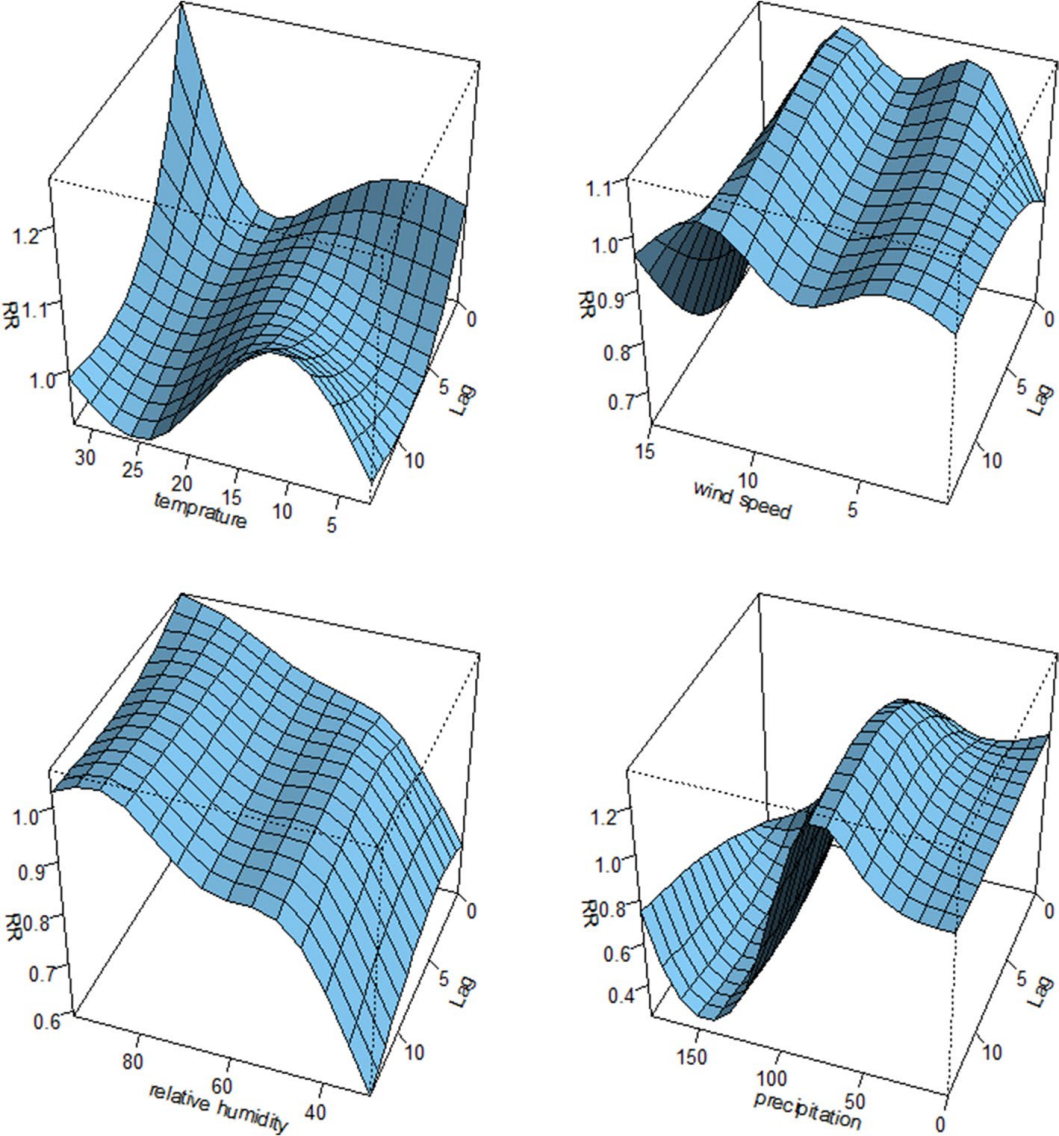
3-D plot of RR along meteorological variables and lags from 2014–2020

**Fig. 3:**
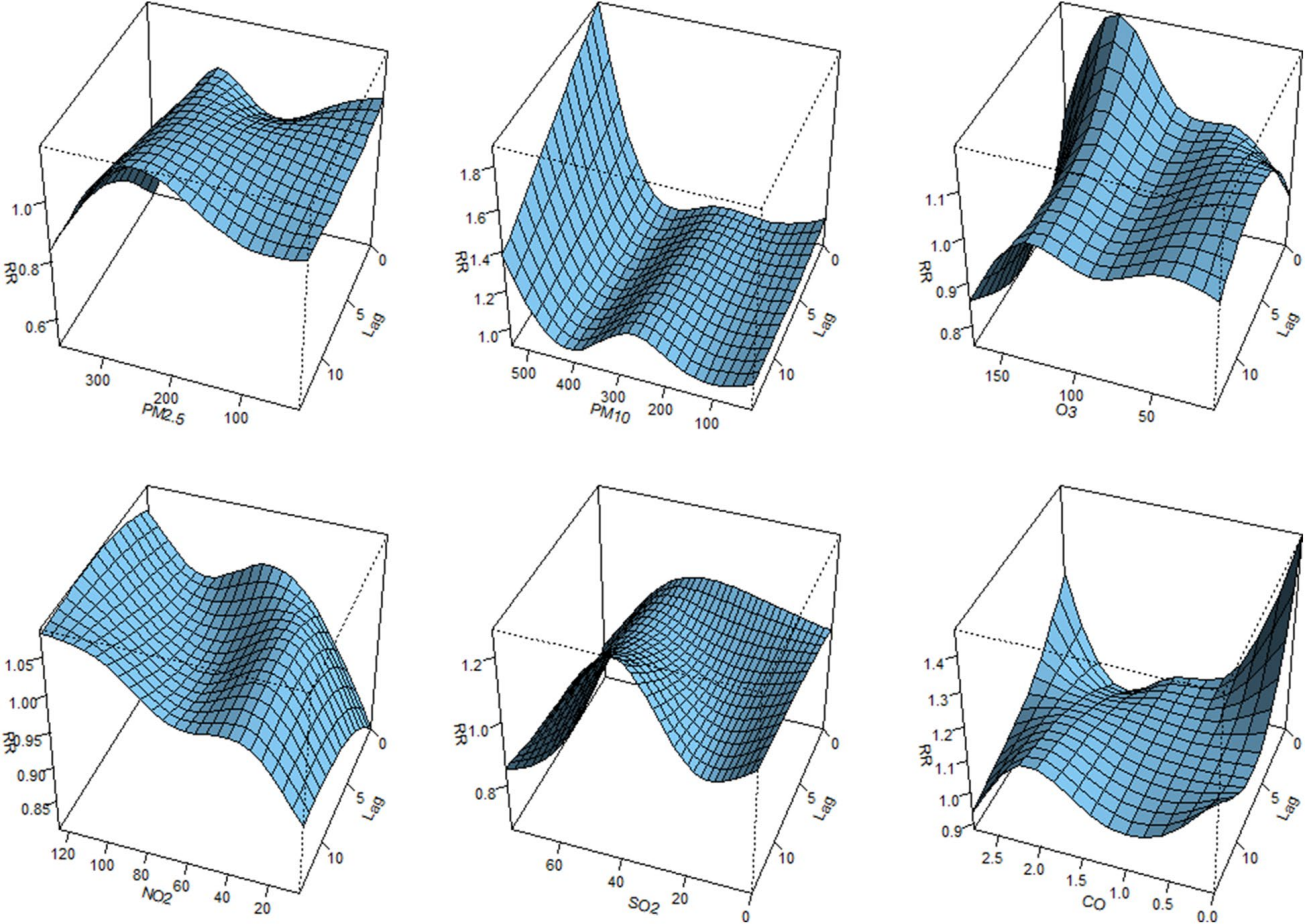
3-D plot of RR along air pollutants and lags from 2014–2020

See as Fig. 2, from 2014–2020, there was a non-linear association between meteorological factors and HFMD incidence. The effect of temperature on HFMD reached its maximum at 32 ℃ at lag 0 days and the association between wind speed and HFMD have a different lag structure. The number of HFMD cases increased with the increase of relative humidity. The relationship between precipitation and HFMD was strongest at 70-80 mm, then gradually decreased and reached the lowest value at 150 mm. Similarly, we found that the effects of different air pollutants on HFMD varied with the lag days and pollutant concentrations, as shown in the Fig. 3. In addition, The 3D plot of the pre-vaccination, post-vaccination and COVID-19 period showed that the lag structure of environmental factors and HFMD in different periods was different. (see Fig. S1, S2, S3, S4, S5, S6).

Different climatic variables and pollutants exerted varied extreme effects on HFMD. It is worth noting that the lag structure of the overall analysis results from 2014 to 2020 shows differences from the three periods, as shown in Table.S4 and Fig.S7, S8, S9, S10.

**Fig. 4:**
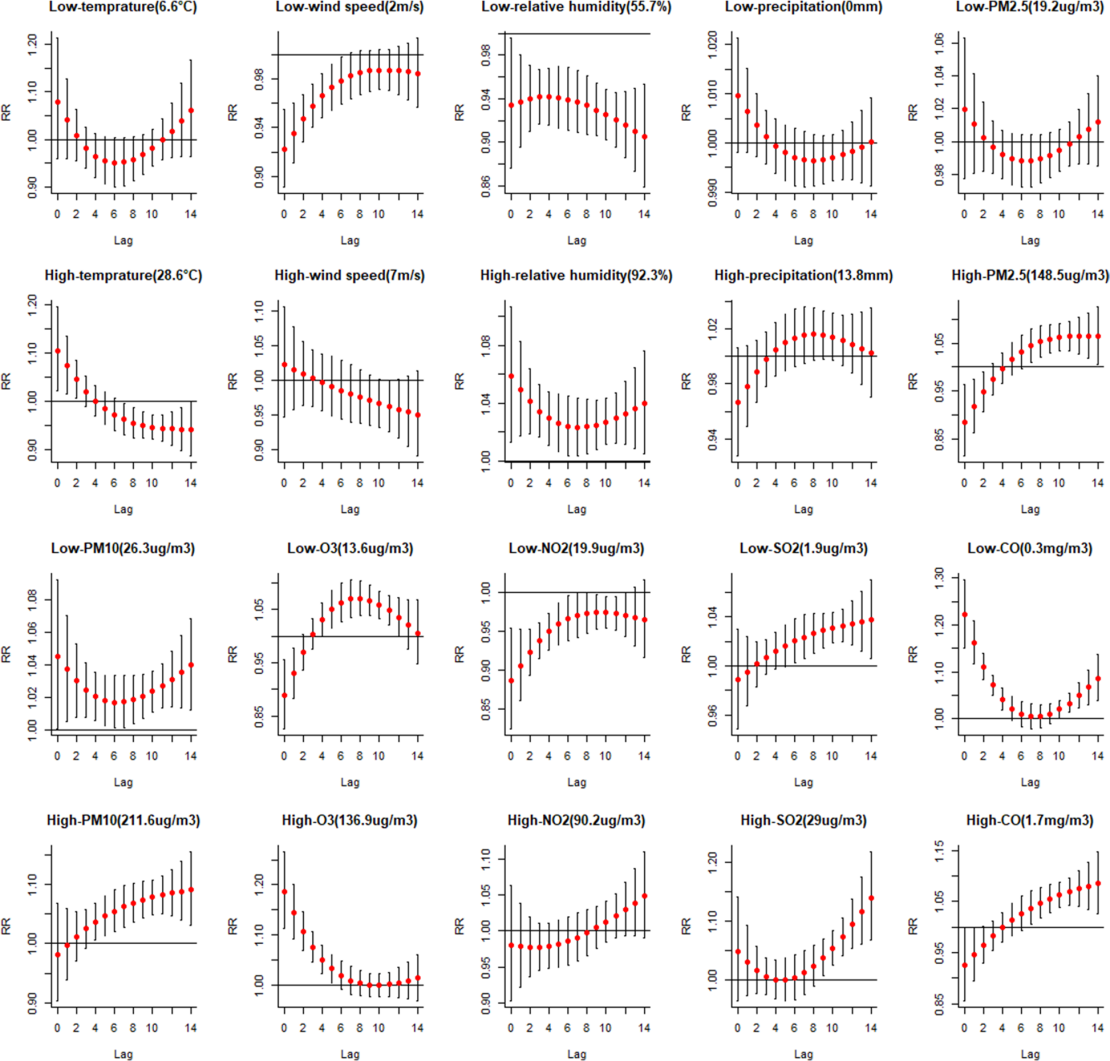
Extreme meteronological and air pollutans variables with HFMD incidence from 2014–2020

See as Fig. 4 and Table.S4, from 2014 to 2020, high relative humidity will significantly increase the risk of HFMD among the meteorological factors. The relationship between high relative humidity and HFMD was v-shaped, with a minimum effect between lag 6–8 days and the cumulative effect was 1.63 (95%CI: 1.39–1.91). Higher precipitation increased the incidence of HFMD, but the effect was not statistically significant. The rest of the studied meteorological factors showed a protective effect, but this protective effect was only statistically significant at low wind speed and relative humidity. See as Fig. 4, the protective effect of low wind speed on HFMD decreased gradually with the increase of lag days, in other words, the protective effect of low wind speed on HFMD was the largest at lag 0 day. Contrastly, the protective effect of low relative humidity on HFMD was strongest at lag 14 days. Among air pollutants, extreme high and low levels of PM_10_, O_3_, SO_2_ and CO will significantly increase the risk of HFMD. In addition, it can be seen from Fig. 4 that except for O_3_, the influence of extremely high concentration of air pollutants on HFMD reached the maximum at lag 14 days. See as Fig. 4, extremely high concentration of PM_2.5_ will significantly increase the incidence of HFMD after lag 6 days, but the cumulative RR (Table S4) is not statistically significant. In addition, we also observed that low level of NO_2_ had a protective effect against HFMD, but this protective effect became weaker as the lag days increased.

Fig.S7, S8, S9, S10 played the relationship between extreme environmental variables and HFMD incidence along lag days in three period. See as Table S4, in pre-vaccination period, extremely high and low temperature and low precipitation can significantly increase the risk of HFMD while low wind speed and relative humidity and high precipitation played a protective role in HFMD incidence. In addition, extremely high concentrations of PM_2.5_, PM_10_, O_3_ and NO_2_ are significantly associated with the incidence of HFMD. Low levels of PM_10_ and NO_2_ will also significantly increase the incidence of HFMD while other air pollutants at low concentrations showed protective effects. In post-vaccination period, high relative humidity and high concentration of O_3_, SO_2_ and CO will increase the risk of HFMD. In COVID-19 period, low temperature and high concentration of PM_2.5_ will increase the incidence of HFMD. Wind speed, PM_2.5_, PM_10_, NO_2_, O_3_ and CO at low level will significantly reduce the risk of HFMD.

Subgroup analysis was conducted to examine the potential effect in terms of gender and age. Consistent with the main analysis, similar associations were observed in both male and female subgroups (Fig S13). But difference associations were obvious in different age groups (Fig. S11, S12). See as Table S5, we found that the cumulative effect of O_3_ and NO_2_ are greatest in children aged 0–1 years old at both extremely low and high concentrations. The effects of low concentrations of SO_2,_ PM_10_ and CO on children aged 6–14 were greater than those of other age groups. Low concentration of PM_2.5_ only had a significant effect on the age group of 6–14 years old, and its cumulative RR was 1.82 (95%CI: 1.56–2.13) while the effect of high concentration of PM_2.5_ on all age groups was not statistically significant. Overall, in addition to high relative humidity and rainfall, the rest of the meteorological factors have a proctive effect to the risk of HFMD for all age groups.

The results obtained were similar to those obtained in sensitivity analyses, which were conducted by adding vaccination population to the model.

## Discussion

In this study, We applied the distributed lag nonlinear model (DLNM) to explore the relationship between climate, air pollution and HFMD incidence in terms of variables and lag days. The results suggest that high relative humidity, high precipitation and extremely high and low levels of PM_10_, O_3_, SO_2_ and CO will increase the risk of HFMD from 2014 to 2020. And high concentration of air pollutants has the greatest impact on 0–1-year-old children.

From 2014 to 2020, we don’t find an association between extremely level of temperature and HFMD. The relationship between temperature and HFMD before the introduction of EV71 vaccine has been explored by many studies and they agreed that temperature changed the incidence of HFMD by affecting the survival and transmission of pathogen as well as human activities and behaviors [21–23]. A study in Guilin [13] found that an extremely low wind speed exerted certain protective effect which were consistent with our research. But study conducted in Hefei indicated that wind speed can increase the risk of HFMD while Huang et. al [24] found no statistically significant association between wind speed and HFMD. This discrepancy may be attributed to the possible confounding effects caused by geographic and socioeconomic distribution. In addition, we found that high relative humidity increased the incidence of HFMD, but this effect was not statistically significant. Numbers of previous studies have found this effect to be meaningful [25–27]. On the one hand, under the condition of high relative humidity, HFMD-related pathogens may be able to thrive depending on humidity, resulting in longer survival times, and have stronger infectiousness [28]. On the other hand, high relative humidity can also limit sweating and then affect the metabolism of children [27]. The correlation between rainfall and HFMD was not found in our study which is consistent with a study in Huainan [29]. It worth noting that precipitation’s values at most days were zero, which could further cause the estimation of exposure–response relationship to progress toward a null value, therefore, different reference values will lead to changes in the impact of precipitation.

Our study found that almost all air pollutants are associated with the risk of HFMD, especially at an extremely high concentration from 2014–2020. Study results show PM_10_ increased the risk of HFMD while PM_2.5_ is not associated with the development of HFMD, which was supported by many studies [13, 19, 30, 31]. The mechanism to explain this relationship between PM_10_ and HFMD is that HFMD is mainly spreads through fecal–oral transmission or through close contact and exposure to air pollution makes children more vulnerable to intestinal infections by hand contact. Thus, HFMD viruses attached to ambient particles may be transported over long distances under favorable weather condition [12, 13]. Gu et al. [15]. found that both moderate and high concentrations of ozone increased the risk of HFMD, and we found that high and low concentration of ozone increased the risk of HFMD from 2014–2020. However, Yu et al. [13]. found that high concentration of O_3_ has a certain protective effect on foot and mouth disease. Thus, we need more research to explore the real relationship between them. We find a significant association between SO_2_ and HFMD from 2014–2020 which supposed by a study in Hefei found that SO_2_ increases the risk of HFMD [11, 32]. Although the mechanism of SO_2_ on hand-foot-mouth disease is not clear, the effect of SO_2_ on respiratory disease has been widely demonstrated [33]. Thus, we consider that SO_2_, like other particulates, affects the immunity of children to increase the risk of enterovirus infection. Our overall analysis suggests that CO increased the risk of HFMD, but studies on CO and HFMD are limited. Yan et al. found a positive effect of CO but insignificant [34]. Although there is no evidence prove that CO is related to the incidence of HFMD, the effect of health is well known. A number of studies demonstrated that chronic CO exposure appears to impart adverse health effects, especially with cardiovascular events [33].

We found that the relationship between environmental factors and HFMD was not consistent before and after the introduction of the vaccine. The independent effects of air pollution and influenza vaccination on childhood HFMD have been extensively investigated, but no study have investigated potential effect modification by vaccination for the relationship between environmental factors and HFMD. A case-crossover study conducted in Taiwan, China [14] and Liu et al. [15]. demonstrated that vaccine might modify the adverse effects of pollutants on some disease. Although the previous studies have different study designs, participant’ characteristics, vaccine types, and health outcomes with our study, they provide indirect support for our findings that vaccine might modify the adverse effects of environmental factors on HFMD. It should be mentioned that we include the number of vaccinations in the sensitivity analysis to consider the effect of collinearity between vaccination and environmental factors. The results suggest that this effect has little effect on the results, which further proves the reliability of our results. Overall, our findings provided the new evidence on supporting the increase in vaccine use for HFMD in Chinese children and adolescents who expose to ambient air pollution.

During the COVID-19 epidemic period, the number of HFMD cases in Chengdu decreased significantly, and the impact of environmental factors on the incidence was not significant. Aside from suspending classes, the government, also took other measures such as closing management in the community, isolating at home and closing all kinds of leisure places, which may reduce contact and airborne diseases [7, 35]. In addition, extremely weather factors and air pollutants have no significant impact on HFMD because of the lack of outdoor activities. It is worth noting that although our results show that there is a correlation between the incidence of HFMD during COVID-19 's period, due to the broad confidence interval and limited sample size, this findings should be interpreted with caution.

In conclusion, our study can only demonstrated that the relationship between HFMD and environmental factors after the introduction of vaccine and COVID-19 epidemic is different from that before vaccine introduction. But whether the relationship was altered by the vaccine and COVID-19 needs to be confirmed by more studies.

The results of stratified analysis showed that Children aged 0–1 years is more affected by high relative humidity due to their immune system is not yet well developed. In addition, the low concentration of air pollutants has the greatest impact on the 6–14 age group, while the high concentration of air pollutants has the greatest impact on the 0–1 age group. Since the age group is analyzed from the overall data, the impact of low concentrations of air pollutants on the 6–14 age group may be attributed to a lack of vaccine protection and more outdoor activities to increase the risk of infection. High concentrations of air pollutants are more likely to attack young children with immature immune mechanisms, thus increasing the risk of the disease. As mentioned above, the goverment should pay more attention to the sensitive group of children when making policies.

There are several limitations to this study. First, cases of negative infection or asymptomatic symptoms may not be included in passive surveillance data, leading to an underestimate of the impact. Second, this study is essentially an ecological study and ecological fallacies are inevitable.

## Conclusion

High relative humidity, high precipitation and extremely levels of PM_10_, O_3_, SO_2_ and CO will increase the risk of HFMD from 2014–2020 and this relationship are different among different ages. The relationship between environmental factors and HFMD after vaccine introduction and during the COVID-19 epidemic was different from that before vaccine introduction. The results of this study provide a reference for local authorities to formulate intervention measures and establish an environment-based disease early warning system.

## Supplementary Information

**Additional file 1: Table S1.** Description of environmental factors in Chengdufrom 2014 to 2016. **Table S2.** Description ofenvironmental factors in Chengdu from 2017 to 2020. **Table S3.** Descriptionof environmental factors in Chengdu in 2020. **Table S4.** The overallestimated RR of different meteorological and air pollution factors in differentyears. **Table S5.** The overall estimated RR of different meteorologicaland air pollutants factors in different age. **Fig S1.** 3-D plot of RRalong meteorological variables and lags in pre-vaccination period. **Fig S2.**3-D plot of RR along air pollutants and lags in pre-vaccination period. **Fig S3.**3-D plot of RR along meteorological variables and lags in post-vaccinationperiod. **Fig S4.** 3-D plot of RR along air pollutants and lags inpost-vaccination period. Fig S5. 3-D plot of RR along meteorological variablesand lags in COVID-19 period. **Fig S6.** 3-D plot of RR along air pollutantsand lags in COVID-19 period. **Fig S7.** Extreme environmental variableswith HFMD incidence in in pre-vaccination period. **Fig S8.** Extremeenvironmental variables with HFMD incidence in post-vaccination period. **Fig S9.**Extreme environmental variables with HFMD incidence in COVID-19 period. **Fig S10.**Extreme meteorological and pollutants variables with HFMD incidence from 2014-2020.**Fig S11.** Low levels of meteorological and pollutants variables with HFMDincidence in different age. **Fig S12.** High levels of meteorological andpollutants variables with HFMD incidence in different age. **Fig S13.**Extreme meteorological and pollutants variables with HFMD incidence indifferent gender.

## Data Availability

Daily meteorological data can download from the publicly accessible China National Weather Data Sharing System (http://data.cma.cn/site/index.html). Daily data of air pollutants were collected from the Sichuan Environmental Monitoring Center (http://sthjt.sc.gov.cn/). The datasets of incidence of HFMD generated during and analyzed during the current study are not publicly available due to restrictions apply to the availability of these data but are available from the corresponding author on reasonable request.

## Abbreviations

HFMD: Hand foot and mouth disease
COVID-19: Coronavirus disease 2019
DLNM: Distributed lag non-linear models
EV71: Enterovirus 71
CVA16: Coxsackievirus A16
CVA6: Coxsackievirus A6
CVA10: Coxsackievirus A10
CDC: Center for Disease Control and Prevention

## References

[1] Head JR, Collender PA, Lewnard JA, Skaff NK, Li L, Cheng Q, Baker JM, Li C, Chen D, Ohringer A (2020). Early Evidence of Inactivated Enterovirus 71 Vaccine Impact Against Hand, Foot, and Mouth Disease in a Major Center of Ongoing Transmission in China, 2011–2018: A Longitudinal Surveillance Study. Clin Infect Dis.

[2] Zhu F, Xu W, Xia J, Liang Z, Liu Y, Zhang X, Tan X, Wang L, Mao Q, Wu J (2014). Efficacy, safety, and immunogenicity of an enterovirus 71 vaccine in China. N Engl J Med.

[3] Li YP, Liang ZL, Xia JL, Wu JY, Wang L, Song LF, Mao QY, Wen SQ, Huang RG, Hu YS (2014). Immunogenicity, safety, and immune persistence of a novel inactivated human enterovirus 71 vaccine: a phase II, Randomized, double-blind, placebo-controlled Trial. J Infect Dis.

[4] Huang W-C, Huang L-M, Kao C-L, Lu C-Y, Shao P-L, Cheng A-L, Fan T-Y, Chi H, Chang L-Y (2012). Seroprevalence of enterovirus 71 and no evidence of crossprotection of enterovirus 71 antibody against the other enteroviruses in kindergarten children in Taipei city. J Microbiol Immunol Infect.

[5] Chen B, Wang M, Huang X, Xie M, Pan L, Liu H, Liu Z, Zhou P (2021). Changes in Incidence of Notifiable Infectious Diseases in China Under the Prevention and Control Measures of COVID-19. Front Public Health.

[6] Sun X, Xu Y, Zhu Y, Tang F (2021). Impact of non-pharmaceutical interventions on the incidences of vaccine-preventable diseases during the COVID-19 pandemic in the eastern of China. Hum Vaccin Immunother.

[7] Niu Y, Luo L, Rui J, Yang S, Deng B, Zhao Z, Lin S, Xu J, Zhu Y, Wang Y (2021). Control measures during the COVID-19 outbreak reduced the transmission of hand, foot, and mouth disease. Journal of Safety Science and Resilience.

[8] Cheng Q, Bai L, Zhang Y, Zhang H, Wang S, Xie M, Zhao D, Su H (2018). Ambient temperature, humidity and hand, foot, and mouth disease: A systematic review and meta-analysis. Sci Total Environ.

[9] Cheng J, Wu J, Xu Z, Zhu R, Wang X, Li K, Wen L, Yang H, Su H (2014). Associations between extreme precipitation and childhood hand, foot and mouth disease in urban and rural areas in Hefei. China Sci Total Environ.

[10] Zhang Z, Xie X, Chen X, Li Y, Lu Y, Mei S, Liao Y, Lin H (2016). Short-term effects of meteorological factors on hand, foot and mouth disease among children in Shenzhen, China: Non-linearity, threshold and interaction. Sci Total Environ.

[11] Wei Q, Wu J, Zhang Y, Cheng Q, Bai L, Duan J, Gao J, Xu Z, Yi W, Pan R (2019). Short-term exposure to sulfur dioxide and the risk of childhood hand, foot, and mouth disease during different seasons in Hefei. China Sci Total Environ.

[12] Chen G, Zhang W, Li S, Williams G, Liu C, Morgan GG, Jaakkola JJK, Guo Y (2017). Is short-term exposure to ambient fine particles associated with measles incidence in China?. A multi-city study Environ Res.

[13] Yu G, Li Y, Cai J, Yu D, Tang J, Zhai W, Wei Y, Chen S, Chen Q, Qin J (2019). Short-term effects of meteorological factors and air pollution on childhood hand-foot-mouth disease in Guilin. China Sci Total Environ.

[14] Huang CH, Chao DY, Wu CC, Hsu SY, Soon MS, Chang CC, Kor CT, Chang WT, Lian IB (2016). Influenza vaccination and the endurance against air pollution among elderly with acute coronary syndrome. Vaccine.

[15] Liu K, Li S, Qian ZM, Dharmage SC, Bloom MS, Heinrich J, Jalaludin B, Markevych I, Morawska L, Knibbs LD (2020). Benefits of influenza vaccination on the associations between ambient air pollution and allergic respiratory diseases in children and adolescents: New insights from the Seven Northeastern Cities study in China. Environ Pollut.

[16] Shakil MH, Munim ZH, Tasnia M, Sarowar S (2020). COVID-19 and the environment: A critical review and research agenda. Sci Total Environ.

[17] Gasparrini A. Distributed Lag Linear and Non-Linear Models in R: The Package dlnm. J Stat Softw. 2011;43(8):1–20. PMC3191524 22003319

[18] Du Z, Lawrence WR, Zhang W, Zhang D, Yu S, Hao Y (2019). Interactions between climate factors and air pollution on daily HFMD cases: A time series study in Guangdong. China Sci Total Environ.

[19] Yin F, Ma Y, Zhao X, Lv Q, Liu Y, Li X, Zhang T (2019). Analysis of the effect of PM10 on hand, foot and mouth disease in a basin terrain city. Sci Rep.

[20] Bhaskaran K, Gasparrini A, Hajat S, Smeeth L, Armstrong B (2013). Time series regression studies in environmental epidemiology. Int J Epidemiol.

[21] Xu Z, Hu W, Jiao K, Ren C, Jiang B, Ma W (2019). The effect of temperature on childhood hand, foot and mouth disease in Guangdong Province, China, 2010–2013: a multicity study. BMC Infect Dis.

[22] Xiao X, Gasparrini A, Huang J, Liao Q, Liu F, Yin F, Yu H, Li X (2017). The exposure-response relationship between temperature and childhood hand, foot and mouth disease: A multicity study from mainland China. Environ Int.

[23] Pearson D, Basu R, Wu XM, Ebisu K (2020). Temperature and hand, foot and mouth disease in California: An exploratory analysis of emergency department visits by season, 2005–2013. Environ Res.

[24] Yong Huang TD (2013). Shicheng Yu, Jing Gu, Cunrui Huang, Gexin Xiao and Yuantao Hao: Effect of meteorological variables on the incidence of hand, foot, and mouth disease in children: a time-series analysis in Guangzhou China. BMC Infect Dis.

[25] Yang H, Wu J, Cheng J, Wang X, Wen L, Li K, Su H (2017). Is high relative humidity associated with childhood hand, foot, and mouth disease in rural and urban areas?. Public Health.

[26] Bo Z, Ma Y, Chang Z, Zhang T, Liu F, Zhao X, Long L, Yi X, Xiao X, Li Z (2020). The spatial heterogeneity of the associations between relative humidity and pediatric hand, foot and mouth disease: Evidence from a nation-wide multicity study from mainland China. Sci Total Environ.

[27] Yang Y, You E, Wu J, Zhang W, Jin J, Zhou M, Jiang C, Huang F (2018). Effects of relative humidity on childhood hand, foot, and mouth disease reinfection in Hefei. China Sci Total Environ.

[28] Luo C, Ma Y, Liu Y, Lv Q, Yin F (2020). The burden of childhood hand-foot-mouth disease morbidity attributable to relative humidity: a multicity study in the Sichuan Basin, China. Sci Rep.

[29] Zhao D, Wang L, Cheng J, Xu J, Xu Z, Xie M, Yang H, Li K, Wen L, Wang X (2017). Impact of weather factors on hand, foot and mouth disease, and its role in short-term incidence trend forecast in Huainan City. Anhui Province International journal of biometeorology.

[30] Liu R, Cai J, Guo W, Guo W, Wang W, Yan L, Ma N, Zhang X, Zhang S. Effects of temperature and PM2.5 on the incidence of hand, foot, and mouth in a heavily polluted area, Shijiazhuang, China. Environ Sci Pollut Res Int. 2022;29(8):11801–14. 10.1007/s11356-021-16397-7 34550518

[31] Huang R, Ning H, He T, Bian G, Hu J, Xu G (2019). Impact of PM(10) and meteorological factors on the incidence of hand, foot, and mouth disease in female children in Ningbo, China: a spatiotemporal and time-series study. Environ Sci Pollut Res Int.

[32] Ji XY, Huang LY, Song J, Fei CN, Liu J, Liu H (2020). Short-term effects of meteorological factors, air pollution, and sunspot on childhood hand, foot, and mouth disease in Tianjin, China: a new time series regression, 2014–2018. Environ Sci Pollut Res Int.

[33] Chen TM, Gokhale J, Shofer S, Kuschner WG (2007). Outdoor air pollution: nitrogen dioxide, sulfur dioxide, and carbon monoxide health effects. Am J Med Sci.

[34] Yan S, Wei L, Duan Y, Li H, Liao Y, Lv Q, Zhu F, Wang Z, Lu W, Yin P (2019). Short-Term Effects of Meteorological Factors and Air Pollutants on Hand, Foot and Mouth Disease among Children in Shenzhen, China, 2009–2017. Int J Environ Res Public Health.

[35] Gupta S, Jawanda MK (2020). The impacts of COVID-19 on children. Acta Paediatr.

