# Supplementary material for: Relationship between meteorological factors, air pollutants and hand, foot and mouth disease from 2014 to 2020
Source: BMC Public Health. 2022 May 17;22:998. doi: 10.1186/s12889-022-13365-9 (PMC9112249; doi:10.1186/s12889-022-13365-9)
Supplement: Supplementary file 1 — Additional file 1: Table S1. Description of environmental factors in Chengdufrom 2014 to 2016. Table S2. Description ofenvironmental factors in Chengdu from 2017 to 2020. Table S3. Descriptionof environmental factors in Chengdu in 2020. Table S4. The overallestimated RR of different meteorological and air pollution factors in differentyears. Table S5. The overall estimated RR of different meteorologicaland air pollutants factors in different age. Fig S1. 3-D plot of RRalong meteorological variables and lags in pre-vaccination period. Fig S2.3-D plot of RR along air pollutants and lags in pre-vaccination period. Fig S3.3-D plot of RR along meteorological variables and lags in post-vaccinationperiod. Fig S4. 3-D plot of RR along air pollutants and lags inpost-vaccination period. Fig S5. 3-D plot of RR along meteorological variablesand lags in COVID-19 period. Fig S6. 3-D plot of RR along air pollutantsand lags in COVID-19 period. Fig S7. Extreme environmental variableswith HFMD incidence in in pre-vaccination period. Fig S8. Extremeenvironmental variables with HFMD incidence in post-vaccination period. Fig S9.Extreme environmental variables with HFMD incidence in COVID-19 period. Fig S10.Extreme meteorological and pollutants variables with HFMD incidence from 2014-2020.Fig S11. Low levels of meteorological and pollutants variables with HFMDincidence in different age. Fig S12. High levels of meteorological andpollutants variables with HFMD incidence in different age. Fig S13.Extreme meteorological and pollutants variables with HFMD incidence indifferent gender. [file 12889_2022_13365_MOESM1_ESM.docx]

**Relationship between meteorological factors, air pollutants and hand, foot and mouth disease from 2014 to 2020**

List of content

Tables

Table.S1:Description of environmental factors in Chengdu from 2014 to 2016

Table.S2:Description of environmental factors in Chengdu from 2017 to 2020

Table.S3:Description of environmental factors in Chengdu in 2020

Table.S4:The overall estimated RR of different meteorological and air pollution factors in different years

Table.S5:The overall estimated RR of different meteorological and air pollutants factors in different age

Figures

Fig.S1 3-D plot of RR along meteorological variables and lags in pre-vaccination period.

Fig.S2 3-D plot of RR along air pollutants and lags in pre-vaccination period.

Fig.S3 3-D plot of RR along meteorological variables and lags in post-vaccination period.

Fig.S4 3-D plot of RR along air pollutants and lags in post-vaccination period.

Fig.S5 3-D plot of RR along meteorological variables and lags in COVID-19 period.

Fig.S6 3-D plot of RR along air pollutants and lags in COVID-19 period.

Fig.S7 Extreme environmental variables with HFMD incidence in in pre-vaccination period.

Fig.S8 Extreme environmental variables with HFMD incidence in post-vaccination period

Fig.S9 Extreme environmental variables with HFMD incidence in COVID-19 period Fig.S10 Extreme meteorological and pollutants variables with HFMD incidence from 2014-2020.

Fig.S11 Low levels of meteorological and pollutants variables with HFMD incidence in different age.

Fig.S12 High levels of meteorological and pollutants variables with HFMD incidence in different age.

Fig. S13 Extreme meteorological and pollutants variables with HFMD incidence in different gender.

| Variables | Mean±SD | Min | P_25_ | Median | P_75_ | Max |
| --- | --- | --- | --- | --- | --- | --- |
| temperature (°C) | 18.13±7.16 | 1.39 | 11.65 | 19.25 | 24.22 | 32.33 |
| Wind speed (m/s) | 3.74±1.58 | 0.98 | 3.00 | 3.00 | 4.16 | 15.02 |
| Relative humidity (%) | 73.99±11.52 | 28.11 | 66.50 | 74.28 | 82.51 | 99.87 |
| Precipitation (mm) | 2.57±8.84 | 0 | 0 | 0 | 1.02 | 121.92 |
| PM_2.5_(ug/m^3^) | 72.65±45.77 | 11.76 | 42.81 | 54.18 | 92.30 | 371.21 |
| PM_10_(ug/m^3^) | 113.0±71.42 | 12.96 | 61.06 | 93.39 | 145.92 | 555.45 |
| O_3_(ug/m^3^) | 72.25±39.86 | 1.86 | 44.50 | 74.25 | 113.70 | 178.48 |
| NO_2_(ug/m^3^) | 59.58±19.53 | 14.49 | 45.53 | 56.94 | 71.07 | 126.49 |
| SO_2_(ug/m^3^) | 16.91±10.65 | 1.83 | 9.29 | 14.57 | 21.27 | 76.42 |
| C0(mg/m^3^) | 1.08±0.43 | 0.21 | 0.80 | 0.99 | 1.29 | 2.90 |

Table.S1:Description of environmental factors in Chengdu from 2014 to 2016

SD: standard deviation; Px: percentile of the data.

Table.S2:Description of environmental factors in Chengdu from 2017 to 2020.

| Variables | Mean±SD | Min | P_25_ | Median | P_75_ | Max |
| --- | --- | --- | --- | --- | --- | --- |
| temperature (°C) | 18.14±7.37 | 1.94 | 11.06 | 18.50 | 24.56 | 31.50 |
| Wind speed (m/s) | 3.83±1.66 | 0.98 | 3.0 | 3.0 | 4.56 | 15.02 |
| Relative humidity (%) | 76.57±11.10 | 29.85 | 69.94 | 77.44 | 85.07 | 99.71 |
| Precipitation (mm) | 3.13±10.37 | 0 | 0 | 0 | 1.27 | 188.47 |
| PM_2.5_(ug/m^3^) | 50.83±33.04 | 3.60 | 26.69 | 42.60 | 57.44 | 272.70 |
| PM_10_(ug/m^3^) | 75.78±50.23 | 8.64 | 41.04 | 63.08 | 99.45 | 434.58 |
| O_3_(ug/m^3^) | 71.70±38.70 | 2.15 | 40.77 | 65.58 | 107.93 | 175.25 |
| NO_2_(ug/m^3^) | 45.28±19.49 | 2.07 | 31.63 | 43.38 | 56.63 | 114.08 |
| SO_2_(ug/m^3^) | 6.28±3.63 | 0 | 3.76 | 5.57 | 7.72 | 26.33 |
| C0(mg/m^3^) | 0.74±0.38 | 0 | 0.51 | 0.70 | 0.88 | 2.65 |

SD: standard deviation; Px: percentile of the data.

Table.S3:Description of environmental factors in Chengdu in 2020

SD: standard deviation; Px: percentile of the data.

| Variables | Mean±SD | Min | P_25_ | Median | P_75_ | Max |
| --- | --- | --- | --- | --- | --- | --- |
| temperature (°C) | 18.35±7.39 | 4.67 | 11.61 | 18.33 | 25.11 | 31.44 |
| Wind speed (m/s) | 3.86±1.68 | 1.07 | 3.00 | 3.35 | 5.01 | 11.98 |
| Relative humidity (%) | 74.56±14.16 | 29.85 | 66.02 | 75.51 | 85.67 | 99.71 |
| Precipitation (mm) | 3.57±15.37 | 0 | 0 | 0 | 1.02 | 188.47 |
| PM_2.5_(ug/m^3^) | 41.70±26.55 | 3.60 | 23.99 | 36.31 | 50.12 | 196.92 |
| PM_10_(ug/m^3^) | 61.06±36.20 | 8.64 | 36.72 | 50.76 | 81.27 | 251.98 |
| O_3_(ug/m^3^) | 72.01±40.62 | 2.25 | 40.49 | 67.06 | 108.37 | 165.64 |
| NO_2_(ug/m^3^) | 33.98±17.06 | 2.07 | 20.38 | 30.86 | 42.36 | 93.15 |
| SO_2_(ug/m^3^) | 4.38±2.32 | 0 | 3.64 | 3.84 | 5.65 | 13.74 |
| C0(mg/m^3^) | 0.61±0.21 | 0 | 0.50 | 0.60 | 0.72 | 1.61 |

Table.S4:The overall estimated RR of different meteorological and air pollution factors in different years

| period | Value | 2014-2016 | 2017-2019 | 2020 | 2014-2020 |
| --- | --- | --- | --- | --- | --- |
| Variables |  | RR (95%CI) | RR (95%CI) | RR (95%CI) | RR (95%CI) |
| Low- temperature | 6.6 | 5.19 (3.33-8.10) | 0.79 (0.40-1.55) | 5.67(3.23-9.96) | 0.94(0.61-1.46) |
| High- temperature | 28.6 | 1.90 (1.42-2.56) | 0.45(0.27-0.74) | 1.86(0.89-3.87) | 0.78(0.58-1.07) |
| Low- Wind speed | 2.0 | 0.63(0.51-0.77) | 0.51 (0.35-0.72) | 0.60(0.46-0.79) | 0.64(0.52-0.79) |
| High- Wind speed | 7.0 | 1.27(0.80-2.01) | 0.19(0.10-0.37) | 0.99(0.47-2.02) | 0.77(0.49-1.19) |
| Low- Relative humidity | 55.7 | 0.59 (0.48-0.73) | 0.04(0.02-0.07) | 0.88(0.41-1.09) | 0.34(0.26-0.43) |
| High- Relative humidity | 92.3 | 1.13(0.95-1.34) | 1.52(1.23-1.87) | 0.83(0.67-1.02) | 1.63(1.39-1.91) |
| Low- Precipitation | 0 | 1.15(1.04-1.27) | 1.05(0.96-1.15) | 1.07(0.93-1.24) | 0.99(0.94-1.06) |
| High- Precipitation | 13.8 | 0.64(0.46-0.88) | 0.86(0.63-1.18) | 0.82(0.51-1.31) | 1.05(0.84-1.29) |
| Low- PM_2.5_ | 19.2 | 1.06(0.86-1.30) | 0.55(0.42-0.72) | 0.51(0.40-0.64) | 0.98(0.85-1.14) |
| High- PM_2.5_ | 148.5 | 2.00(1.51-2.64) | 0.59(0.22-1.54) | a | 1.24(0.90-1.71) |
| Low- PM_10_ | 26.3 | 1.32(1.13-1.55) | 1.11(0.79-1.54) | 0.55(0.41-0.725) | 1.50(1.30-1.73) |
| High- PM_10_ | 211.6 | 2.61(2.09-3.26) | 0.30(0.14-0.62) | b | 2.12(1.59-2.84) |
| Low- O_3_ | 13.6 | 0.95(0.77-1.19) | 0.63(0.43-0.93) | 0.66(0.53-0.81) | 1.32(1.04-1.69) |
| High- O_3_ | 136.9 | 1.54(1.23-1.94) | 1.07(0.73-1.57) | 1.47(0.87-2.49) | 1.86(1.45-2.39) |
| Low- NO_2_ | 19.9 | 3.42(2.18-5.36) | 0.38(0.30-0.49) | 1.11(0.84-1.47) | 0.48(0.41-0.58) |
| High- NO_2_ | 90.2 | 1.63(1.20-2.21) | 0.28(0.16-0.49) | 0.09(0.01-0.53) | 0.99(0.72-1.38) |
| Low- SO_2_ | 1.9 | 0.88(0.76-1.02) | 0.46(0.37-0.57) | 1.38(0.92-2.07) | 1.33(1.23-1.44) |
| High- SO_2_ | 29 | 1.06(0.86-1.30) | 5.48(0.02-1500.11) | 1.13(0.63-2.05) | 1.89(1.49-2.38) |
| Low- C0 | 0.3 | 0.46(0.34-0.62) | 3.98(3.35-4.73) | 0.54(0.41-0.71) | 1.42(1.09-1.13) |
| High- C0 | 1.9 | 1.19(0.87-1.64) | 0.45(0.20-1.01) | c | 2.36(2.08-2.69) |

a:173.06(5.62-5325.68); b:31911.04(0.44-2292847000); c: 0.08(0.0002-43.1698)

Table.S5 The overall estimated RR of different meteorological and air pollutants factors in different age

| Variables | Value | 0–1 year old | 2–3 years old | 4–5 years old | 6-14 years old |
| --- | --- | --- | --- | --- | --- |
| Low- temperature | 6.6 | 0.40(0.25-0.65) | 0.96(0.62-1.47) | 0.79(0.53-1.17) | 0.83(0.51-1.34) |
| High- temperature | 28.6 | 1.14(0.82-1.58) | 0.99(0.72-1.35) | 0.91(0.66-1.25) | 0.71(0.50-1.02） |
| Low- Wind speed | 2 | 0.57(0.46-0.71) | 0.60(0.49-0.73) | 0.70(0.58-0.86) | 0.56(0.45-0.70) |
| High- Wind speed | 7 | 0.85(0.54-1.36) | 0.67(0.43-1.04) | 0.74(0.49-1.14) | 0.98(0.60-1.61) |
| Low-Relative humidity | 56.5 | 0.56(0.43-0.74) | 0.47(0.36-0.60) | 0.41(0.32-0.51) | 0.24(0.18-0.31) |
| High-Relative humidity | 92.3 | 2.12(1.81-2.49) | 1.54(1.31-1.82) | 1.50(1.27-1.76) | 1.48(1.22-1.79) |
| Low- Precipitation | 0 | 0.98(0.93-1.04) | 0.98(0.93-1.04) | 0.99(0.93-1.06) | 0.93(0.87-1.01) |
| High- Precipitation | 14 | 1.11(0.95-1.38) | 1.10(0.89-1.36) | 1.05(0.83-1.33) | 1.27(0.98-1.64) |
| Low- PM_2.5_ | 19.2 | 1.04(0.90-1.21) | 0.95(0.82-1.09) | 1.17(0.99-1.36) | 1.82(1.56-2.13) |
| High- PM_2.5_ | 150.4 | 1.27(0.90-1.80) | 1.33(0.97-1.82) | 1.07(0.78-1.47) | 0.69(0.48-1.00) |
| Low- PM_10_ | 27 | 1.45(1.25-1.68) | 1.36(1.18-1.56) | 1.90(1.63-2.21) | 2.53(2.18-2.93) |
| High- PM_10_ | 211.6 | 2.00(1.45-2.75) | 2.15(1.62-2.85) | 1.62(1.21-2.17) | 1.04(0.75-1.45) |
| Low- O_3_ | 13.5 | 1.41(1.08-1.83) | 1.22(0.95-1.56) | 1.33(1.04-1.69) | 1.03(0.76-1.39) |
| High- O_3_ | 135.6 | 2.21(1.71-2.86) | 1.73(1.35-2.22) | 1.79(1.40-2.28) | 1.04(0.79-1.37) |
| Low- NO_2_ | 20.1 | 0.36(0.29-0.43) | 0.51(0.43-0.61) | 1.16(1.01-1.34) | 1.66(1.44-1.91) |
| High- NO_2_ | 90.5 | 0.90(0.63-1.28) | 0.94(0.68-1.30) | 1.07(0.79-1.47) | 0.41(0.28-0.61) |
| Low- SO_2_ | 1.9 | 1.44(1.32-1.56) | 1.33(1.23-1.44) | 1.33(1.23-1.44) | 1.91(1.74-2.10) |
| High- SO_2_ | 29.1 | 1.72(1.33-2.22) | 2.24(1.79-2.81) | 1.48(1.18-1.86) | 0.90(0.68-1.18） |
| Low- C0 | 0.3 | 3.69(3.26-4.19) | 1.91(1.75-2.08) | 1.30(1.18-1.42) | 1.59(1.37-1.84) |
| High- C0 | 1.7 | 1.72(1.30-2.27) | 1.49(1.17-1.91) | 1.15(0.90-1.45) | 1.10(0.82-1.48) |


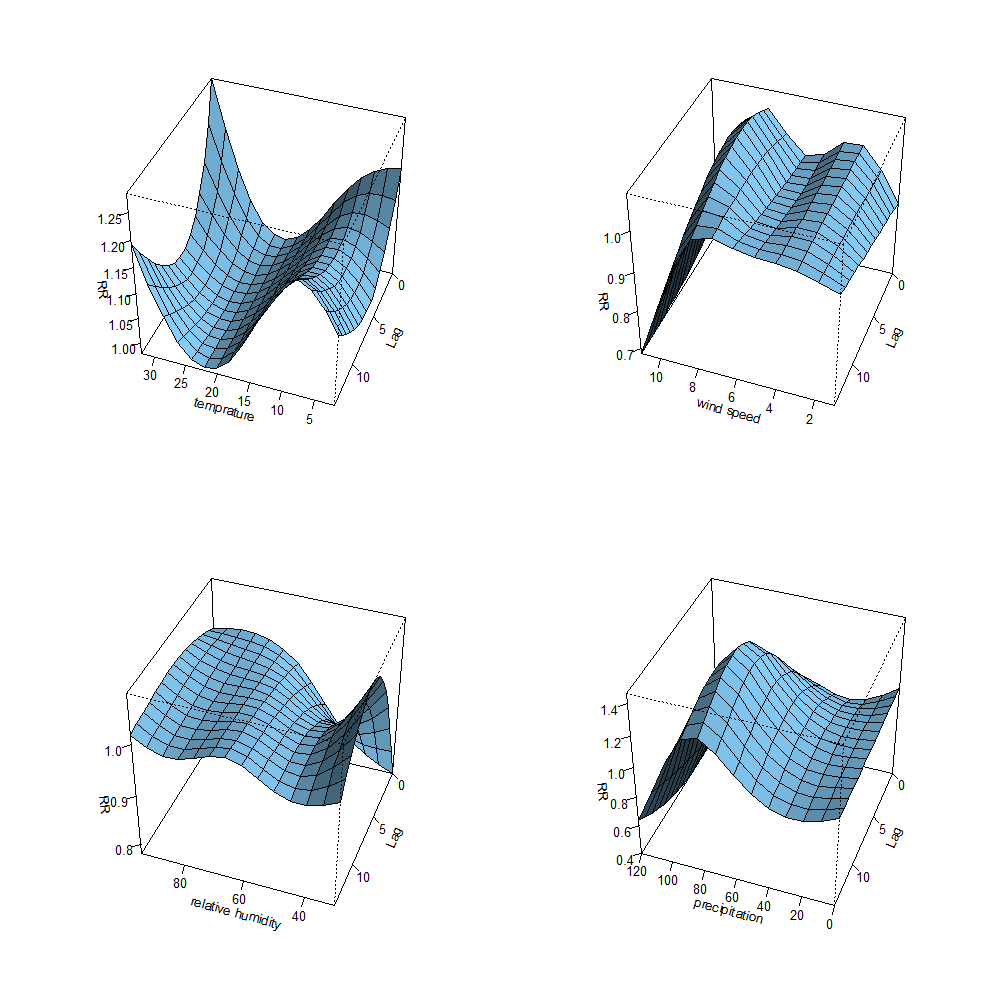
 Fig.S1 3-D plot of RR along meteorological variables and lags in pre-vaccination period.


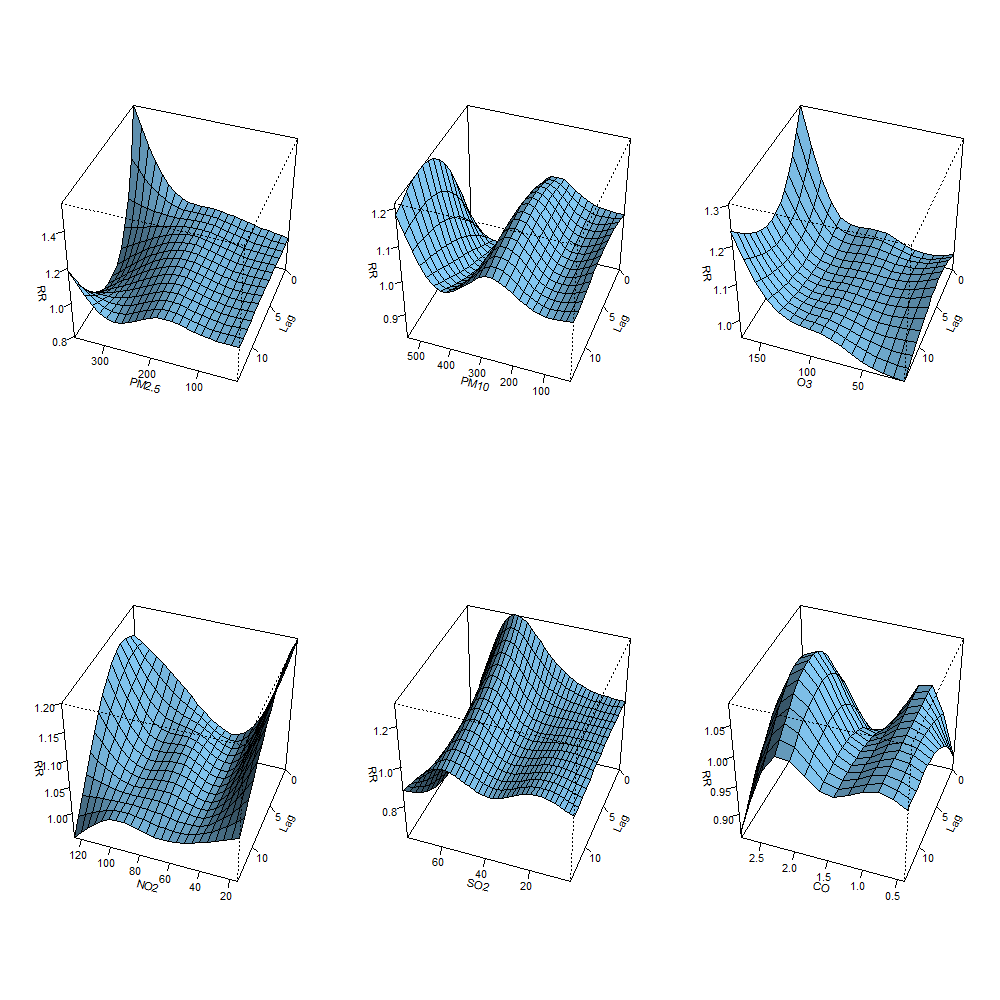
 Fig.S2 3-D plot of RR along air pollutants and lags in pre-vaccination period.


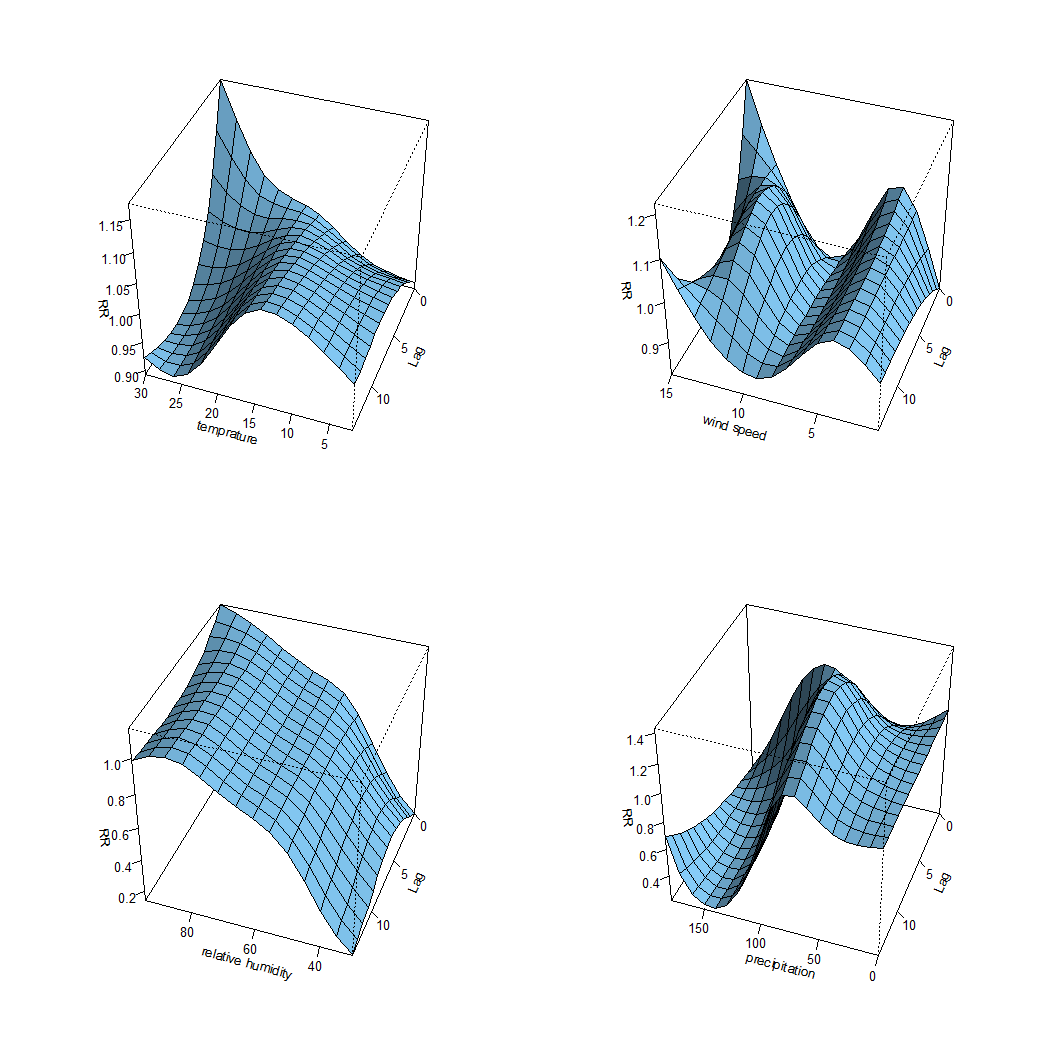
 Fig.S3 3-D plot of RR along meteorological variables and lags in post-vaccination period.


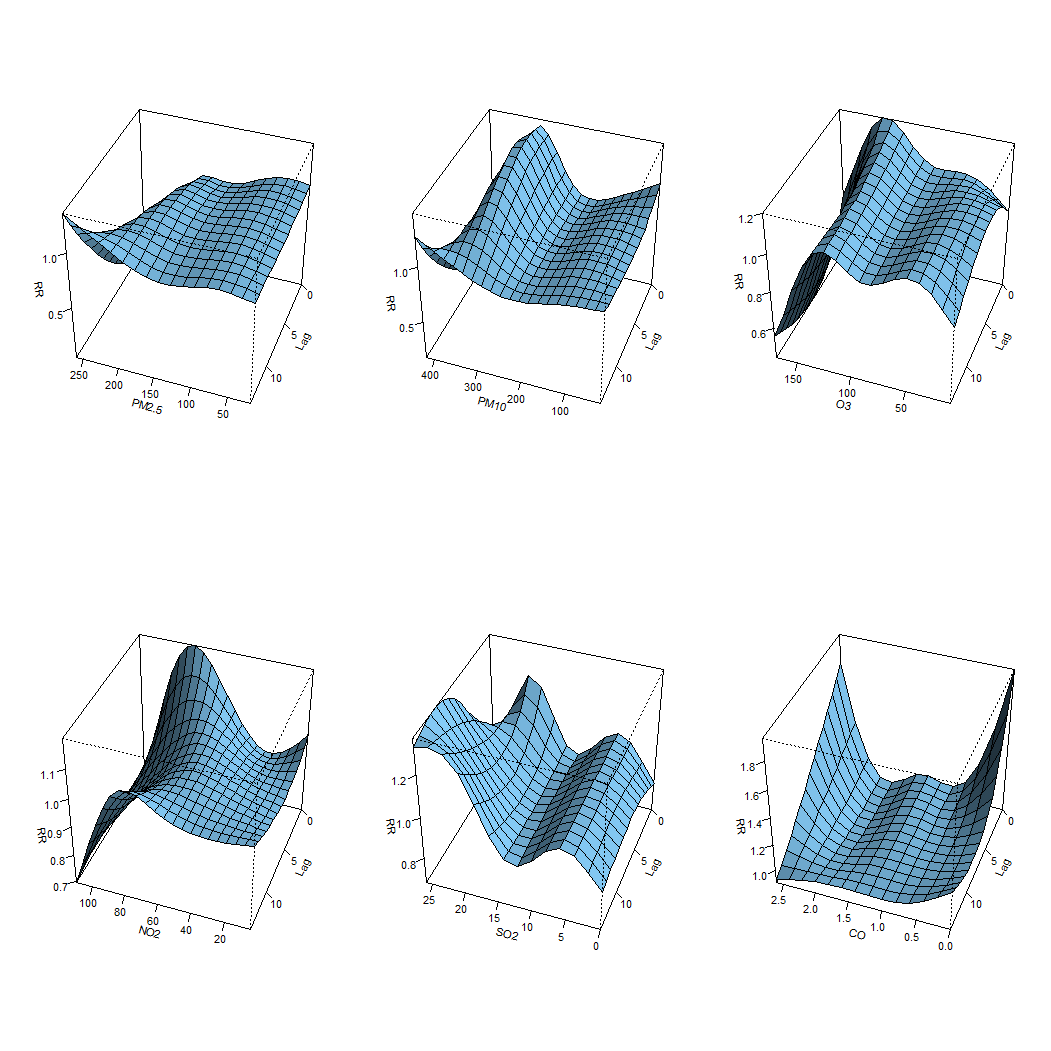
 Fig.S4 3-D plot of RR along air pollutants and lags in post-vaccination period.


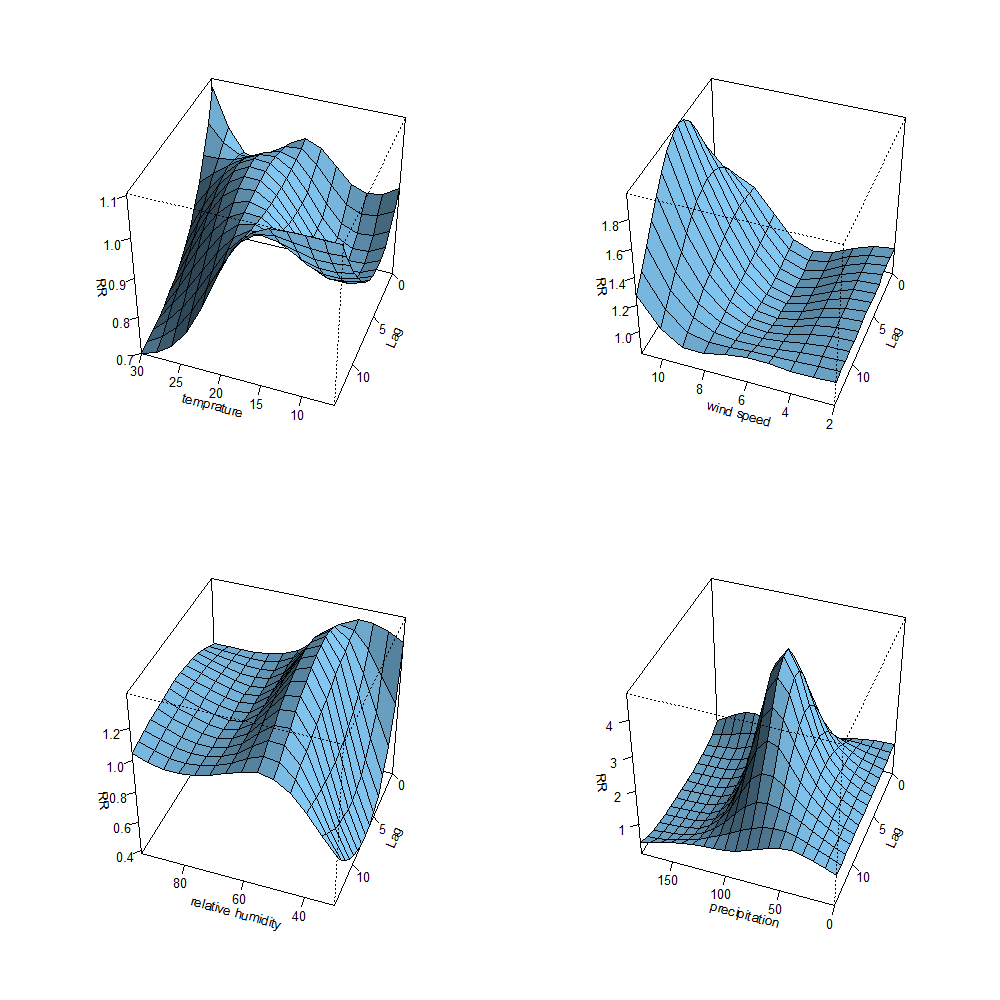
 Fig.S5 3-D plot of RR along meteorological variables and lags in COVID-19 period.


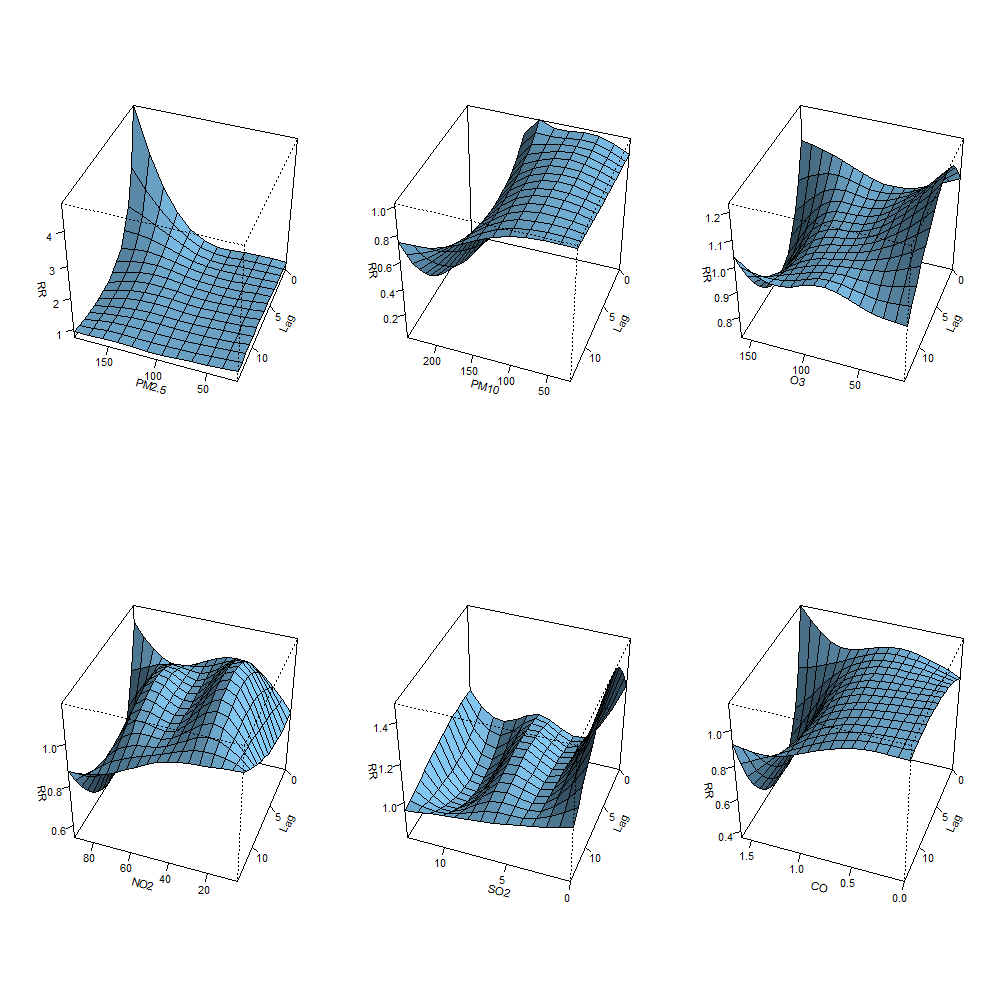
 Fig.S6 3-D plot of RR along air pollutants and lags in COVID-19 period.





Fig.S7 Extreme environmental variables with HFMD incidence in pre-vaccination period.


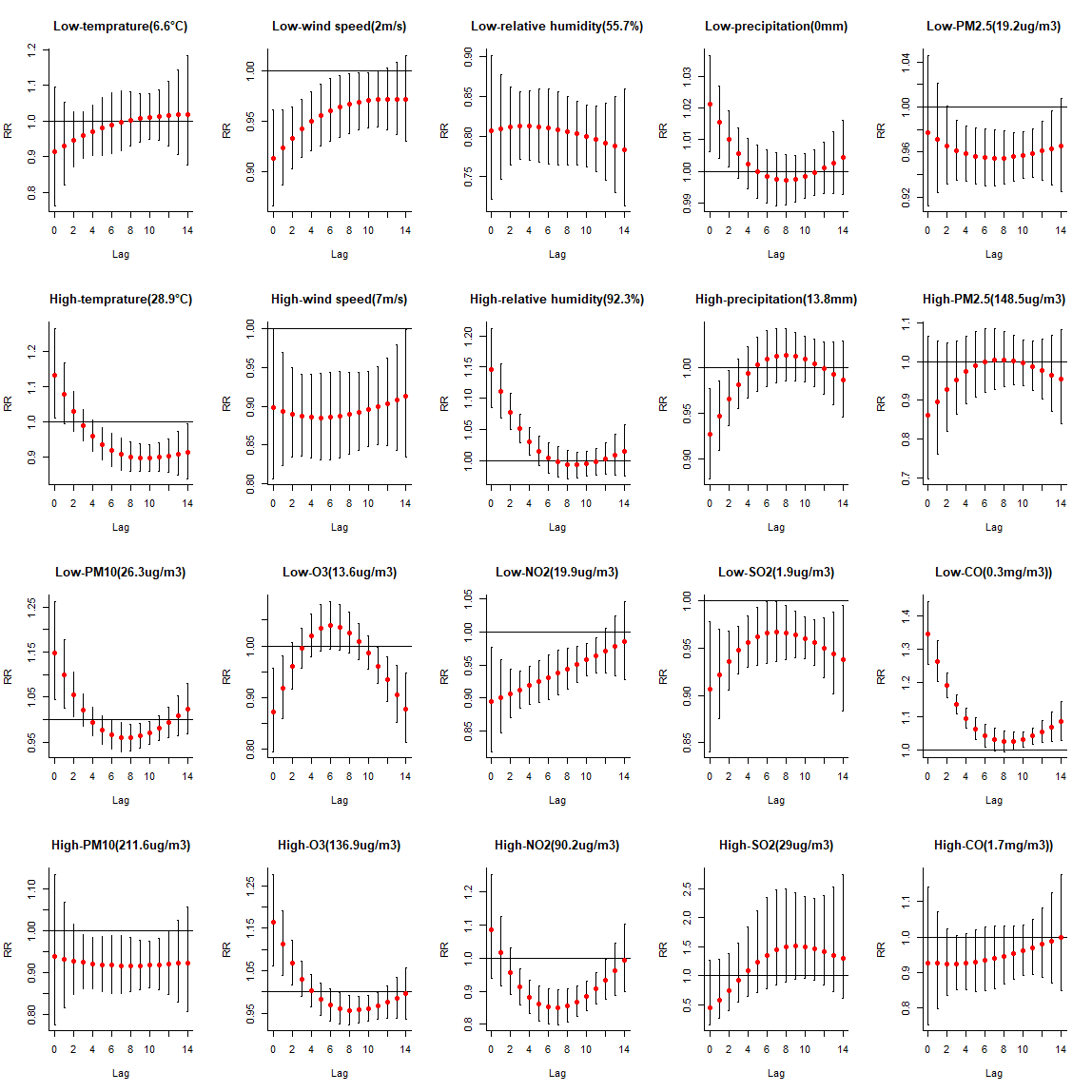


Fig.S8 Extreme environmental variables with HFMD incidence in post-vaccination period.


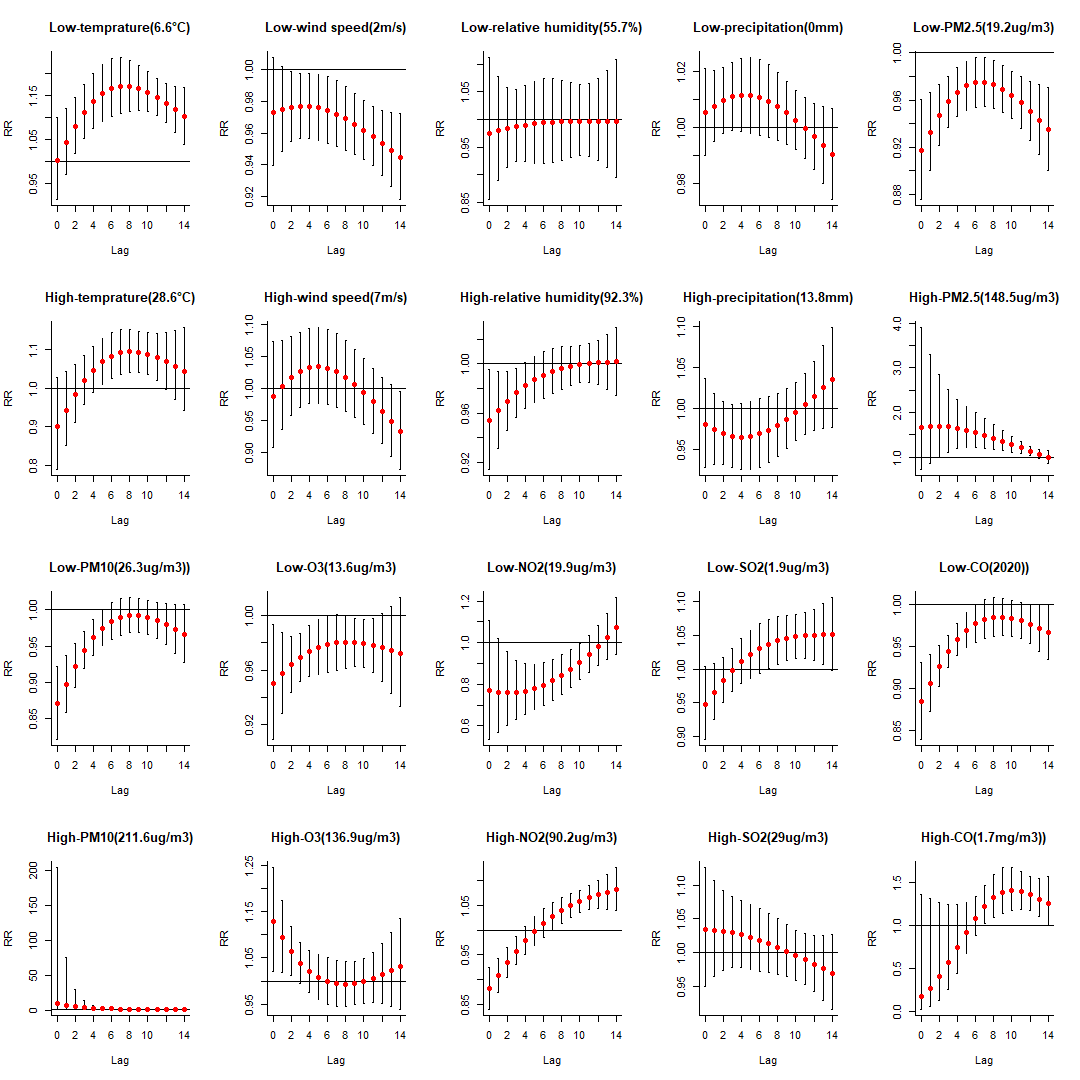


Fig.S9 Extreme environmental variables with HFMD incidence in COVID-19 period.


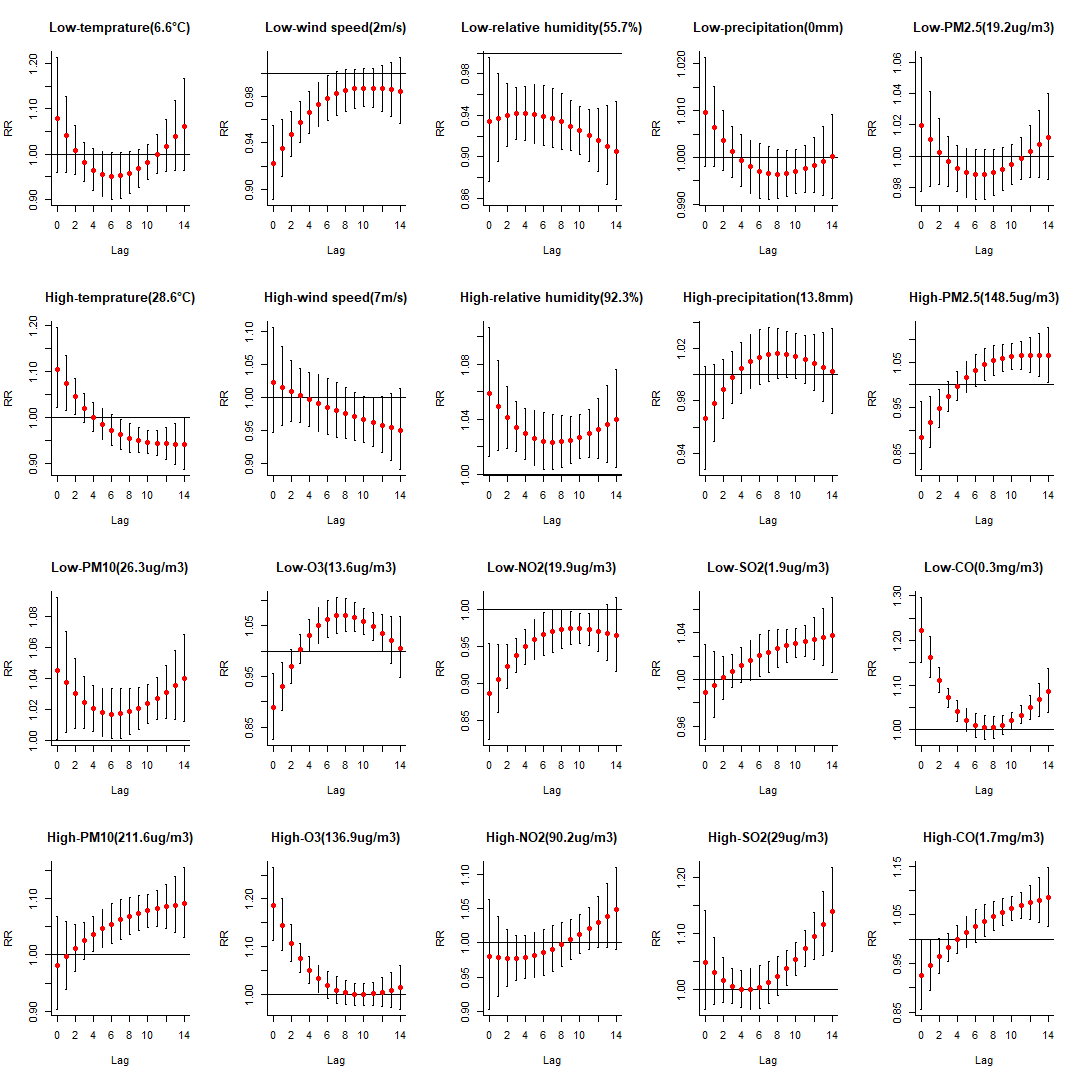


Fig.S10 Extreme meteorological and pollutants variables with HFMD incidence from 2014-2020


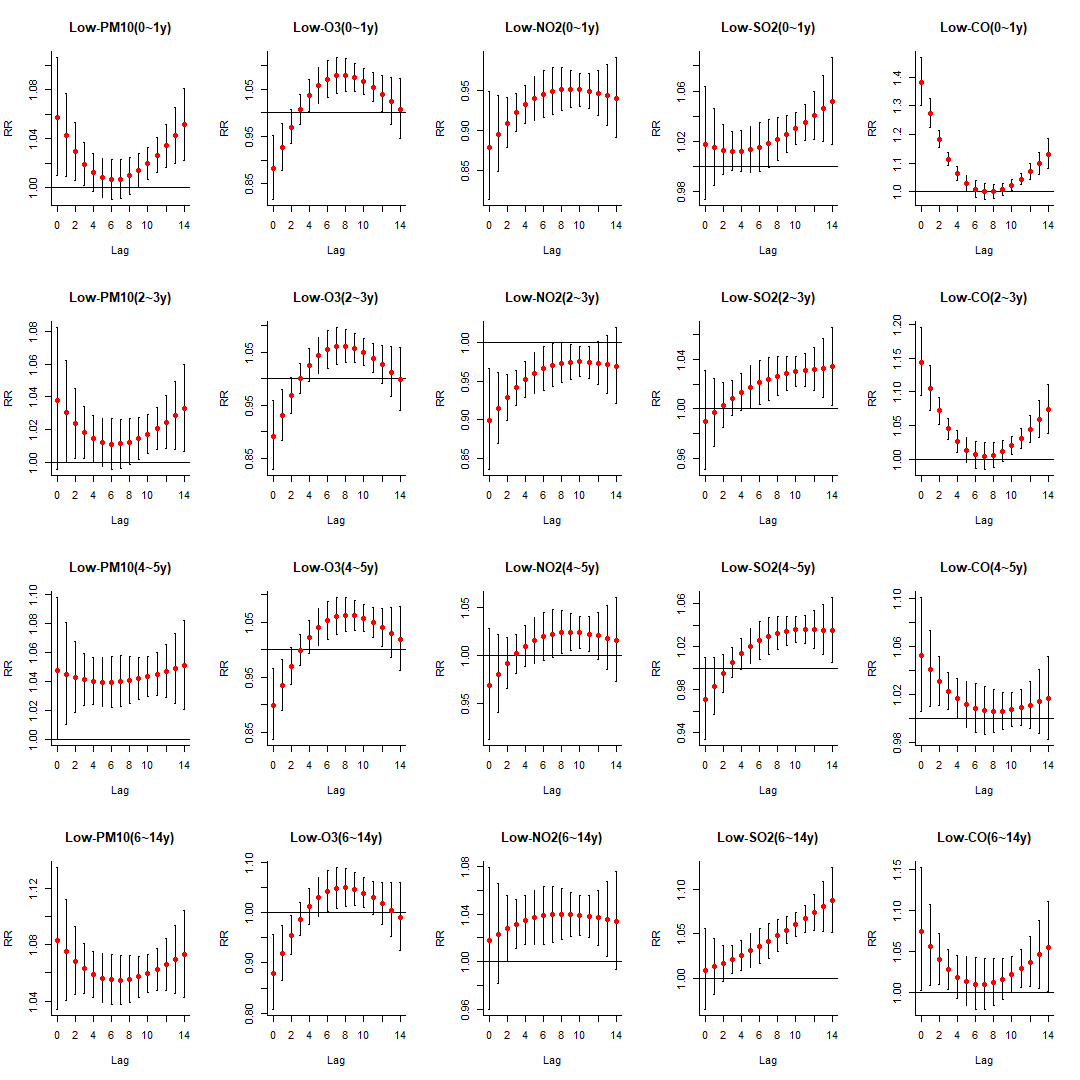

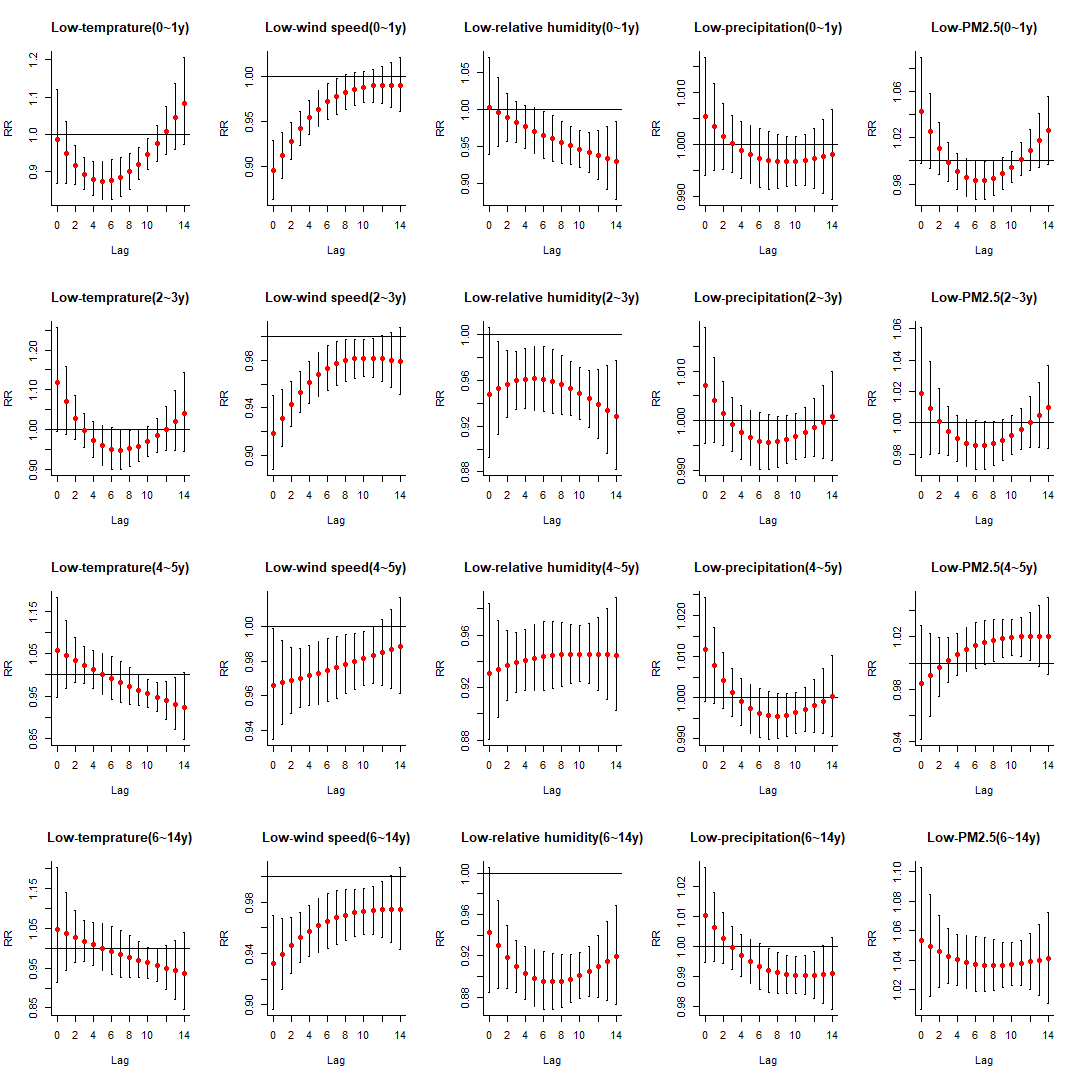
Fig.S11 Low levels of meteorological and pollutants variables with HFMD incidence in different age.


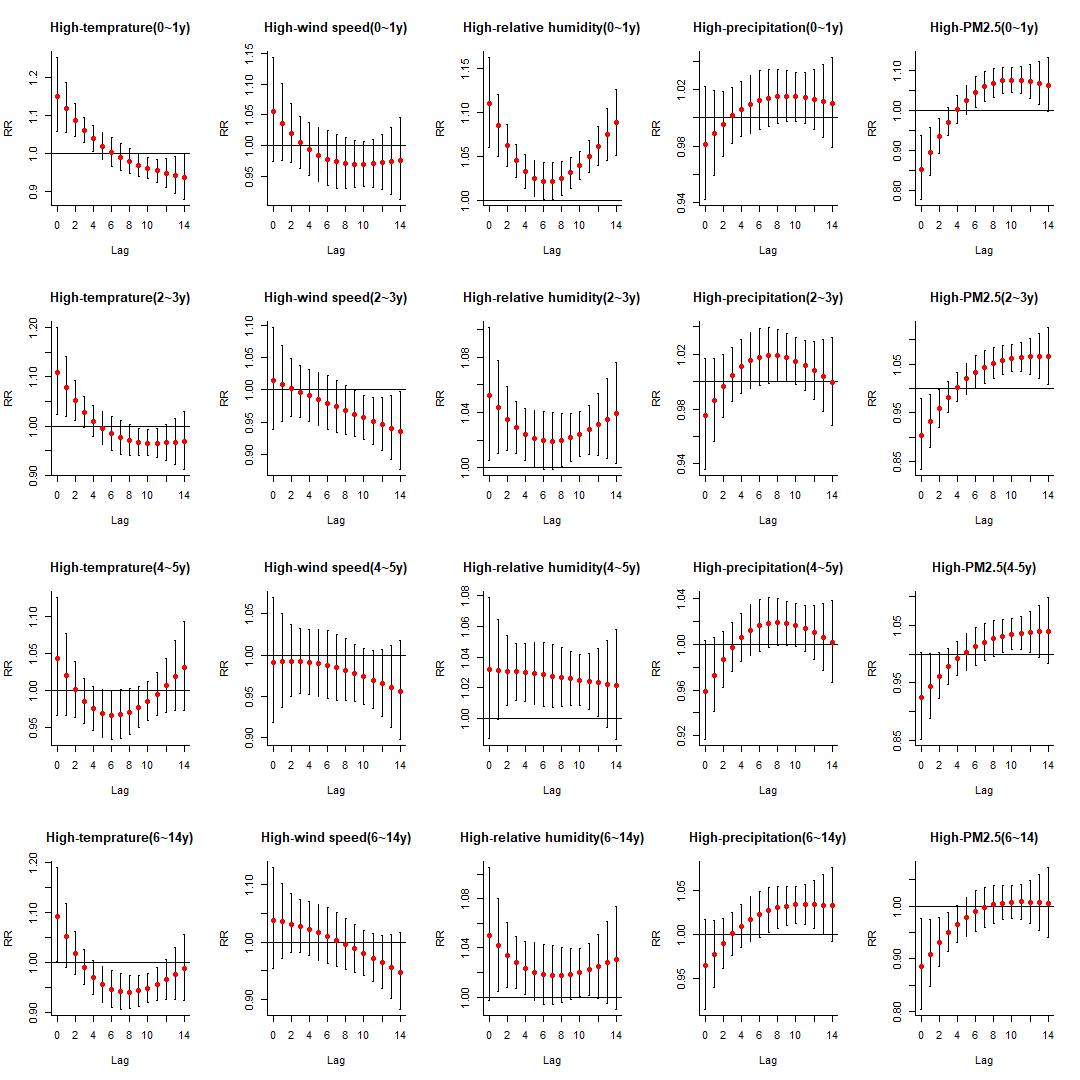


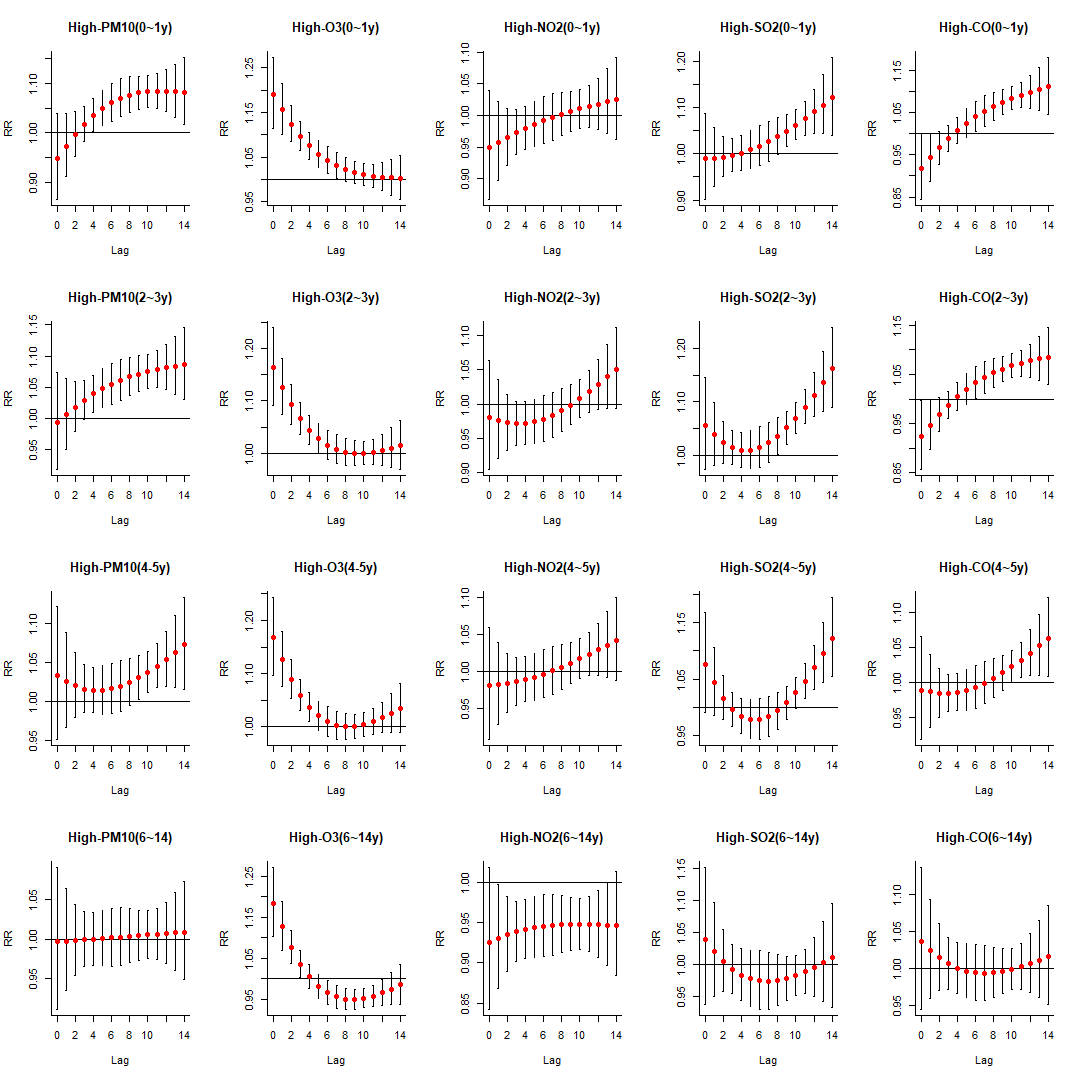


Fig.S12 High levels of meteorological and pollutants variables with HFMD incidence in different age.


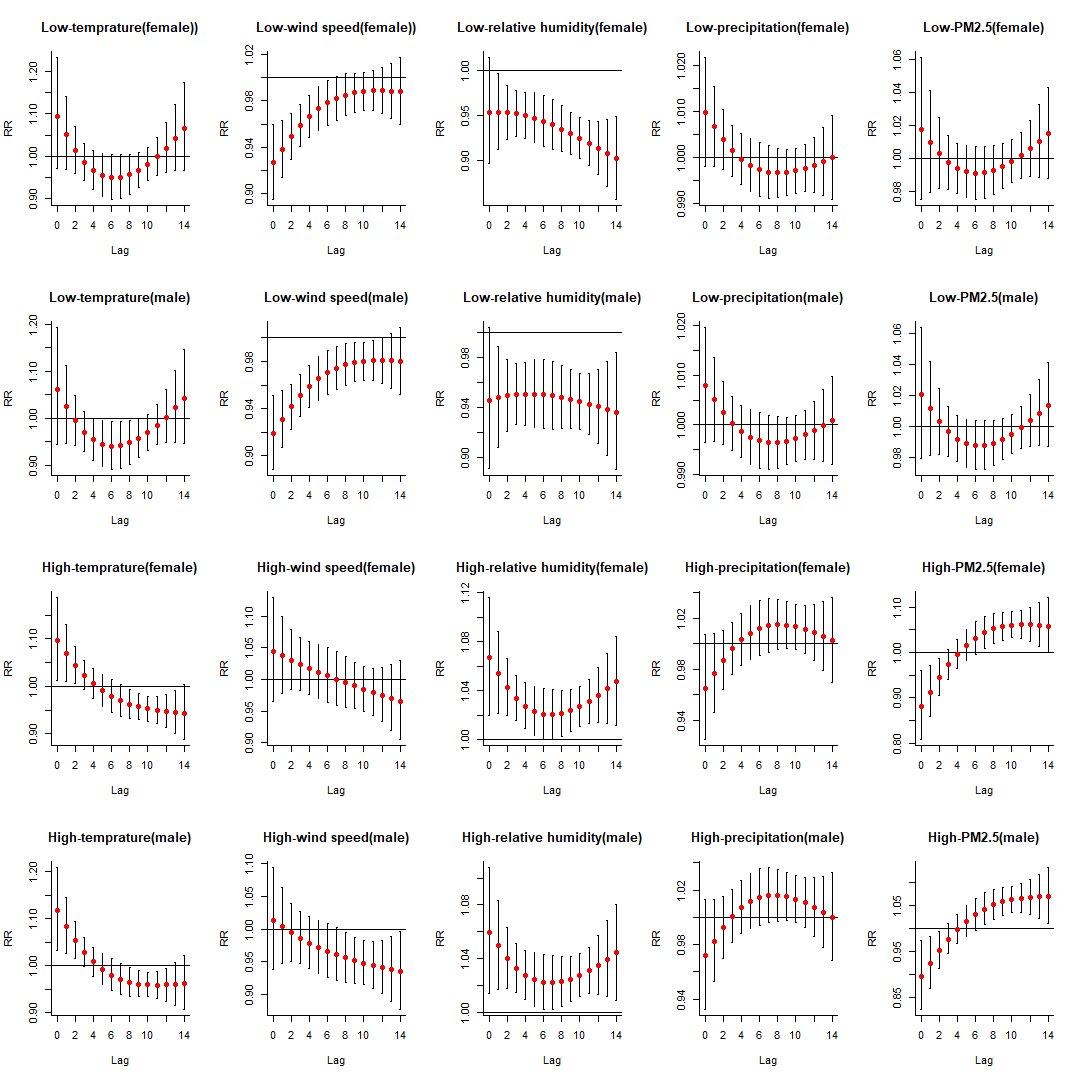


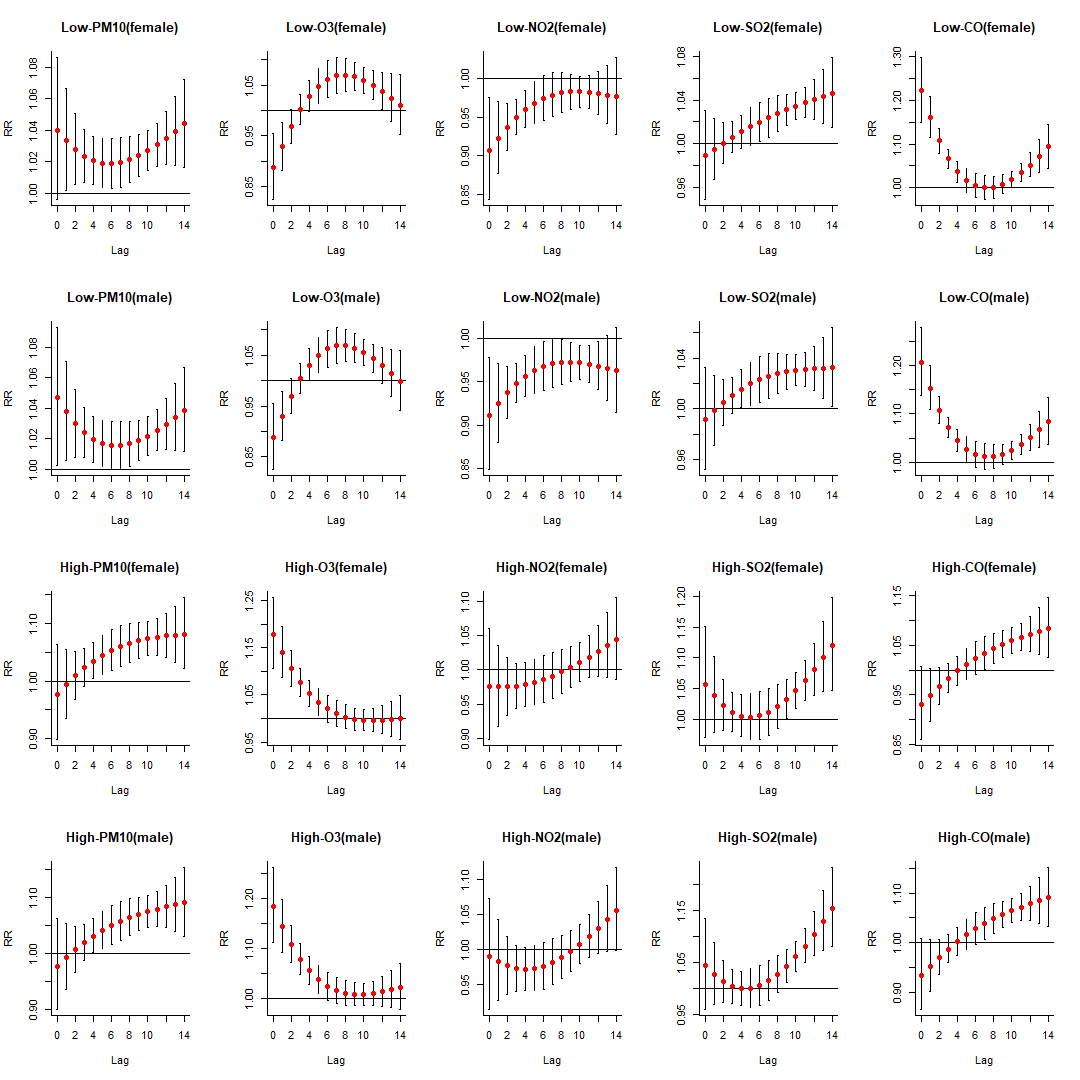


Fig. S13 Extreme meteorological and pollutants variables with HFMD incidence in different gender.
